# Archaeogenetic insights into the demographic history of Late Neanderthals

**DOI:** 10.1073/pnas.2520565123

**Published:** 2026-03-23

**Authors:** Charoula M. Fotiadou, Jesper Borre Pedersen, Hélène Rougier, Mirjana Roksandic, Maria A. Spyrou, Kathrin Nägele, Ella Reiter, Hervé Bocherens, Andrew W. Kandel, Miriam N. Haidle, Timo P. Streicher, Nicholas J. Conard, Flora Schilt, Ricardo Miguel Godinho, Thorsten Uthmeier, Luc Doyon, Patrick Semal, Johannes Krause, Alvise Barbieri, Dušan Mihailović, Isabelle Crevecoeur, Cosimo Posth

**Affiliations:** ^a^Archaeo- and Palaeogenetics, Institute for Archaeological Sciences, Department of Geosciences, University of Tübingen, Tübingen 72074, Germany; ^b^Senckenberg Centre for Human Evolution and Palaeoenvironment at the University of Tübingen, Tübingen 72074, Germany; ^c^Research Center “The Role of Culture in Early Expansions of Humans (ROCEEH)” of the Heidelberg Academy of Sciences and Humanities, University of Tübingen, Tübingen 72074, Germany; ^d^Department of Anthropology, California State University Northridge, Northridge, CA 91330; ^e^Natural Sciences and Engineering Research Council of Canada, Canada Research Chair in Human Evolution, Department of Anthropology, University of Winnipeg, Winnipeg MB R3T 3C7, Canada; ^f^HUMAN ORIGINS—Cluster of Excellence for Integrative Human Origins Studies (EXC 3101), University of Tübingen, Tübingen 72074, Germany; ^g^Department of Archaeogenetics, Max Planck Institute for Evolutionary Anthropology, Leipzig 04103, Germany; ^h^Biogeology, Department of Geosciences, University of Tübingen, Tübingen 72074, Germany; ^i^Department of Early Prehistory and Quaternary Ecology, University of Tübingen, Tübingen 72070, Germany; ^j^Department of Art and Culture, History and Antiquity, Vrije Universiteit Amsterdam, Amsterdam 1081 HV, The Netherlandsl; ^k^Interdisciplinary Center for Archaeology and the Evolution of Human Behavior (ICArEHB), Faculdade das Ciências Humanas e Sociais, Universidade do Algarve (UAlg), Campus de Gambelas, Faro 8005-139, Portugal; ^l^Friedrich-Alexander-Universität Erlangen-Nürnberg (FAU), Department of Classical World and Asian Cultures, Institute of Prehistory and Protohistory, Erlangen 91054, Germany; ^m^De la Préhistorie à l’Actuel: Culture, Environnement et Anthropologie, CNRS, Université de Bordeaux, Ministère de la Culture, Pessac 33615, France; ^n^Service of Scientific Heritage, Royal Belgian Institute of Natural Sciences, Brussels 1000, Belgium; ^o^Department of Archaeology, Faculty of Philosophy, University of Belgrade, Belgrade 11000, Serbia

**Keywords:** neanderthals, ancient DNA, archaeology, demographic history

## Abstract

The demographic history of Neanderthals is only partially understood. In Europe, some degree of genetic continuity has been shown from 120 thousand years ago (ka) onward despite the occurrence of multiple subsequent diversification events. While it has been proposed that a population turnover preceded the emergence of Late Neanderthals in Europe, the extent, timing, and geographic location of this event are currently unknown. Here, we report ten mitochondrial DNA sequences (mtDNAs) of Neanderthal individuals from six archaeological sites in Belgium, France, Germany and Serbia, and analyze them alongside 49 published mtDNAs. The integration of phylogenetic and molecular dating analyses with an extensive archaeological dataset enabled us to reconstruct temporal and spatial patterns in Neanderthal distribution. Remarkably, nearly all Late Neanderthal individuals across Europe belong to a single mtDNA lineage that diversified recently, confirming a large-scale genetic replacement. Our analyses date this diversification event to approximately 65 ka and suggest that it likely originated from a population refugium in southwestern France from which Neanderthals appear to have undergone a major range dispersal across Europe. In addition, we detect a sharp decline in the Neanderthal mtDNA effective population size beginning ~45 ka and reaching a minimum ~42 ka, shortly before their extinction. This study demonstrates that integrating molecular and archaeological datasets provides a more detailed understanding of the Late Neanderthal population’s history, and highlights the critical role of climate-driven refugia and subsequent range expansions in shaping the genetic landscape of Neanderthals through time.

## Significance

Knowledge of the population history of Neanderthals remains incomplete, including the evolutionary processes that preceded their extinction. This study provides evidence for a widespread genetic replacement in the demographic history of European Neanderthals. By integrating mtDNA and archaeological data, we reveal a geographic contraction followed by the expansion of Late Neanderthal populations, likely influenced by climatic fluctuations and resulting in a high genetic homogeneity before their disappearance. Specifically, our analyses suggest that Late Neanderthals across Europe largely derive from a major diversification event that took place ~65 ka in southwestern France. This was followed by a wider geographic spread, which is consistent with a postglacial population re-expansion across Europe.

The genetic history of Neanderthals is poorly understood since only a limited number of Neanderthal remains have been genetically investigated. Studies of mitochondrial DNA (mtDNA) have been pivotal in unraveling the Neanderthal evolutionary history. Early analyses of the hypervariable region of the mtDNA revealed that Neanderthals fall outside the genetic variation of modern humans (1) and that they were distributed not only in western Eurasia but also as far east as the Altai mountains in southern Siberia (2). The advent of next generation sequencing allowed for the reconstruction of the first complete Neanderthal mtDNA, unequivocally confirming that the sequenced Neanderthal mtDNA represented an outgroup to modern humans (3). With the development of targeted enrichment techniques, additional complete Neanderthal mtDNA genomes became available from across Europe, consistently showing low genetic diversity and suggesting a smaller effective population size compared to modern humans (4, 5). In 2010, the mitochondrial genome of an unknown hominin group was sequenced, determining Neanderthals and modern humans as a sister group compared to the newly sequenced group named Denisovans (6). In the same year, the first draft nuclear genomes of both Neanderthals and Denisovans were published (7, 8). In addition to revealing evidence of gene flow from Neanderthals into present-day non-Africans and from Denisovans into present-day Oceanians, it was shown that both archaic hominin groups are more closely related to each other on the nuclear genome level than to modern humans. The genomic sequencing of Middle Pleistocene hominins from Sima de los Huesos in Spain (~430 ka) added further complexity to the picture, revealing a nuclear genome resembling early Neanderthals alongside a mtDNA signal akin to Denisovans (9, 10). This discrepancy was attributed to gene flow from an early modern human group into Neanderthals, a scenario first supported by mtDNA (11), and later corroborated with Y-chromosome analyses (12) and more recent genome-wide studies (13, 14).

The analysis of sediment samples from Paleolithic caves has significantly expanded the recovery of hominin mitochondrial and nuclear DNA, even in the absence of skeletal remains (15–17). Nuclear DNA retrieved from Middle Paleolithic sediments at Galería de las Estatuas in Spain, revealed evidence of at least two Neanderthal population radiations, dated to ~135 and ~105 ka (15). In contrast, ancient genomic studies from the Altai mountains indicate a population replacement by western Neanderthal groups between 120 and 80 ka (18, 19). Meanwhile, genetic data from European Neanderthals show a substantial degree of continuity from roughly 120 to 40 ka and spanning Marine Isotope Stages (MIS) 5-3 (20). However, a population turnover also appears to have occurred toward the end of Neanderthal presence in Europe. Late Neanderthals, associated with MIS 3 and dated to 57 to 40 ka, were found to be more closely related to one another than to earlier Neanderthal populations from the same regions (21).

These demographic patterns—particularly population contractions, re-expansions, and lineage replacements across Europe—as well as their temporal and geographic spans, remain poorly understood. Furthermore, the integration of genetic data with archaeological evidence to contextualize these population dynamics has been limited so far. In this study, we provide a demographic perspective on the emergence and development of Late Neanderthals based on ten newly generated mtDNA genomes examined alongside a comprehensive dataset of an additional 49 Neanderthal mtDNA sequences. Our results reveal a geographic contraction followed by a re-expansion of Neanderthals across Europe, characterized by lineage replacements preceding their final disappearance. To contextualize these genetic signals, we integrated an extensive archaeological dataset capturing diachronic shifts in material culture across western Eurasia. We show that the genetic replacement preceding the Late Neanderthal diversification coincided with a reduced distribution of archaeological assemblages. Integrating cultural and genetic evidence interpreted alongside the climatic record, this interdisciplinary analysis offers a more nuanced understanding of the demographic shifts experienced by Neanderthals leading up to their extinction.

## Results

### Archaeological Contexts and Data Generation.

**Fig. 1.**
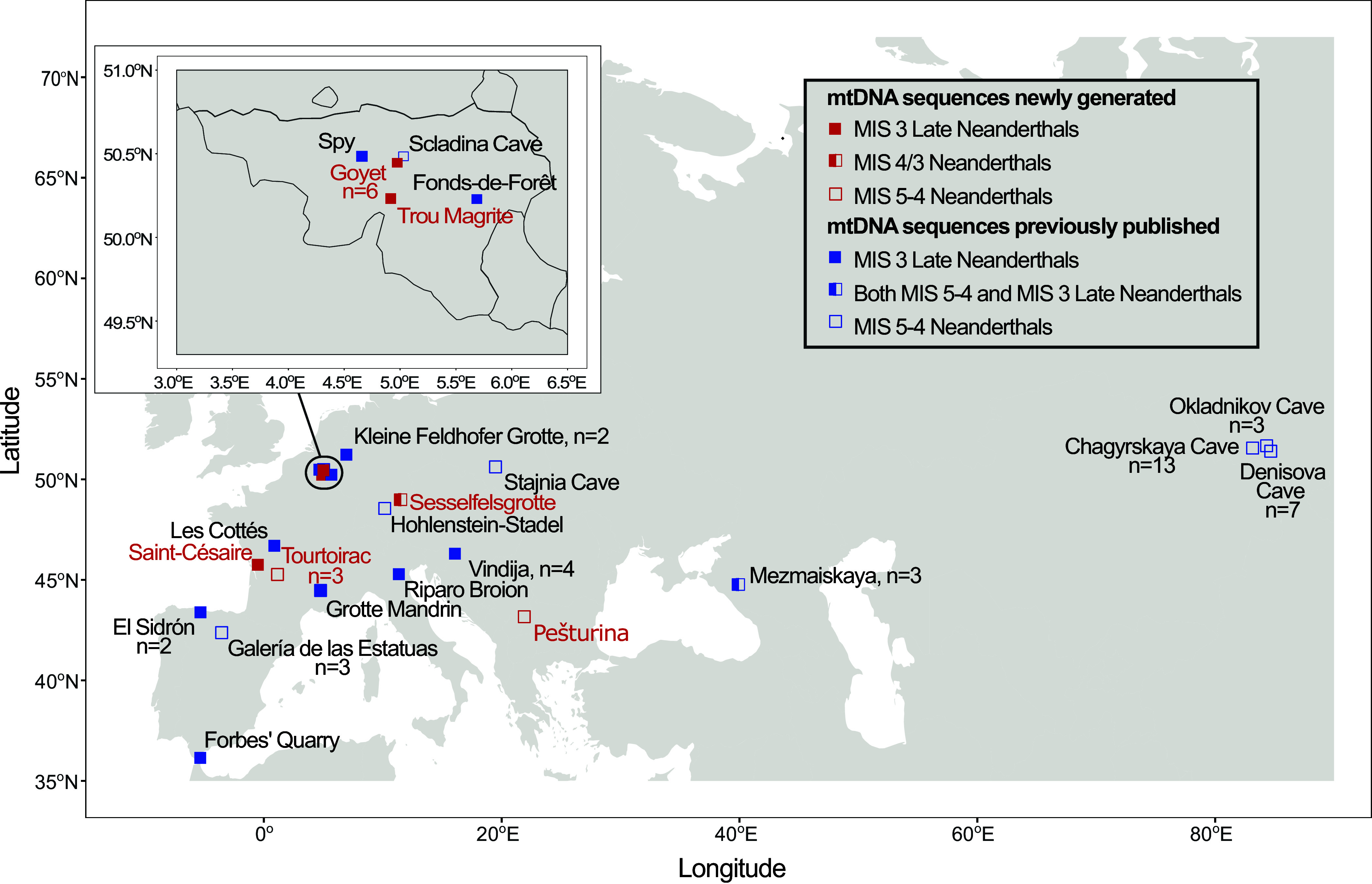
Archaeological sites from which Neanderthal mtDNA sequences have been reported with at least 85% covered positions. Sites with mtDNAs generated in this study and previously published mtDNAs from MIS 5-4 and MIS 3 Neanderthals are depicted as indicated in the legend. For sites that have yielded mtDNA for more than one individual, their numbers (n) are provided below or next to the site names.

We generated ten complete or partial Neanderthal mitochondrial genomes from six archaeological sites in Belgium, France, Germany, and Serbia (Fig. 1 and *SI Appendix*, Table S1). These include three sequenced Late Neanderthal individuals from the Troisième caverne of Goyet in Belgium (*SI Appendix*, Figs. S1–S3), where both nuclear genomes and mtDNA sequences from six Neanderthal remains had previously been recovered (5, 21). We also report genetic data from a Late Neanderthal neonate found in Mousterian layers at La Roche-à-Pierrot, Saint-Césaire, France (*SI Appendix*, Fig. S5) (22). Additional mtDNA data were obtained from a Neanderthal neonate at Trou Magrite in Belgium (*SI Appendix*, Fig. S4), one of two known Neanderthal remains from the site, although its chronological and cultural context remains uncertain (23, 24). Another mitochondrial genome was recovered from a Neanderthal fetus at the Sesselfelsgrotte rock shelter in Germany (*SI Appendix*, Fig. S10), whose exact dating is also uncertain (25). Furthermore, we analyzed three pre-MIS 3 Neanderthals from Tourtoirac, France (*SI Appendix*, Figs. S6–S8), associated with a Quina-type Mousterian context (26). Last, we generated mtDNA data from a pre-MIS 3 Neanderthal tooth found at the Pešturina cave site in Serbia (*SI Appendix*, Fig. S9) (27). Further information on the genetically analyzed specimens and associated archaeological sites is reported in the *SI Appendix*, section 1.

All specimens were micro-computed tomography scanned prior to sampling between 8 and 77 mg of bone or tooth powder, from which DNA was extracted and converted into single-stranded genetic libraries (28, 29). We performed target enrichment of the entire mtDNA using in-solution capture (30, 31), and sequenced the resulting libraries on next generation sequencing platforms. The mean coverage ranged between 14 and 146-fold and the C-to-T deamination at the 5’-end between 10% and 58% (*SI Appendix*, Table S1 and section 2 and Dataset S1). We estimated mtDNA contamination levels (32) and filtered the data from individuals with evidence of present-day contamination above 10% based on the presence of the typical ancient DNA damage (*SI Appendix*, Table S2) (33). Finally, we assembled mtDNA consensus sequences (*Materials and Methods* and *SI Appendix*, section 3) and used them for phylogenetic, molecular dating, and demographic analyses.

### Phylogenetic Analysis.

**Fig. 2.**
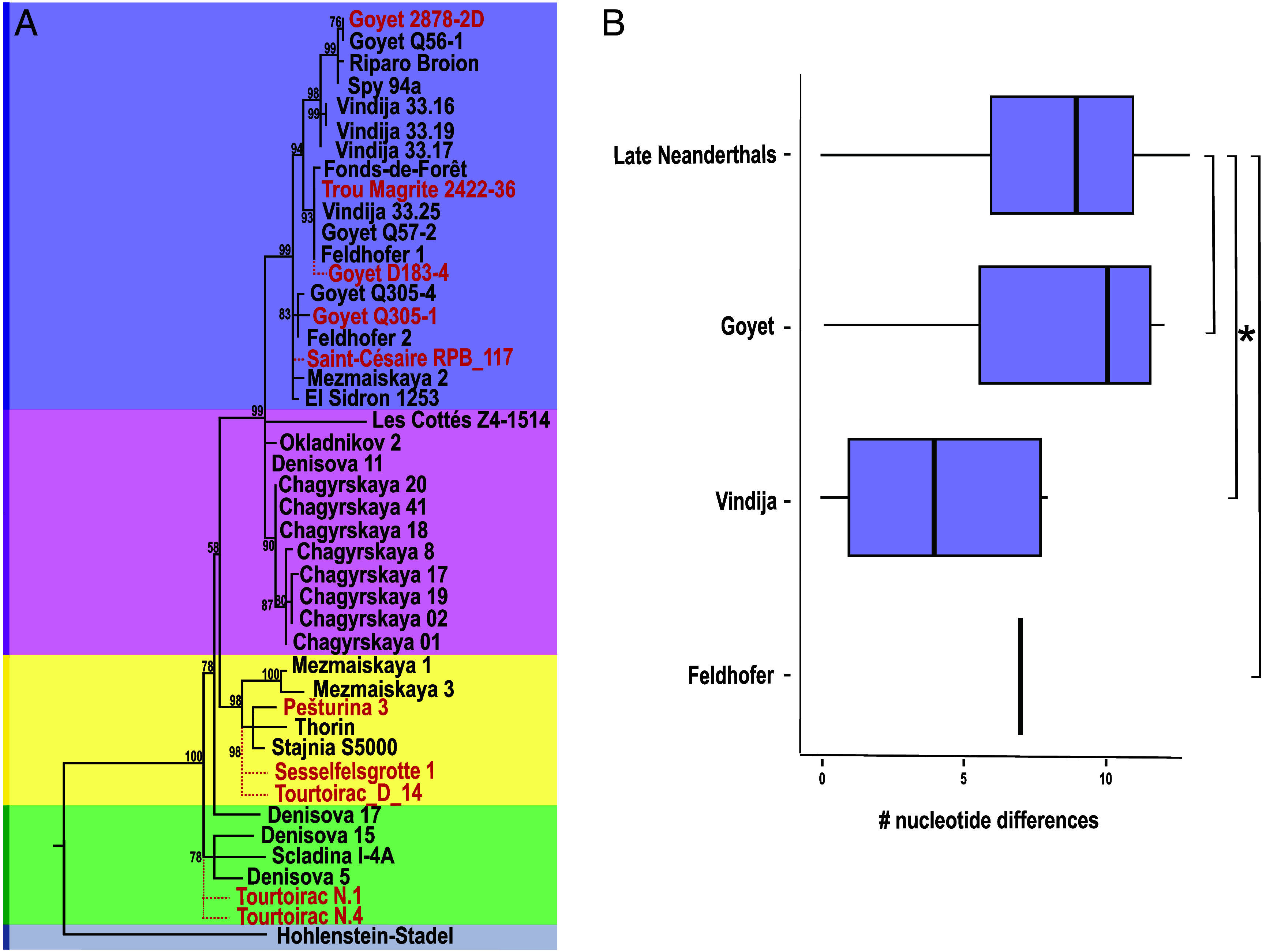
(*A*) Maximum Parsimony tree built with the coding region of the mtDNA from 34 previously published (in black) and four newly generated (in red) Neanderthals (rCRS used as an outgroup). The tree was created with complete deletion and 500 bootstrap iterations (bootstrap support reported on major nodes). The phylogenetic placements of Goyet D138-4, Saint-Césaire RPB_117, Tourtoirac N.1, N.4, D_14 and Sesselfelsgrotte 1 are shown with dotted red lines and are based on a separate SNP analysis (*SI Appendix*, section 4). (*B*) Boxplot of the nucleotide distance for the coding region among mtDNAs within the main Late Neanderthal branch and three archaeological sites from which more than one Late Neanderthal mtDNA are reported. A Wilcoxon rank sum test revealed no significant difference with Goyet and Feldhofer (adjusted *P* = 0.638), and a marginally significant difference with Vindija (adjusted *P* = 0.065).

Phylogenetic analyses of the newly generated mitochondrial genomes alongside previously published nearly complete Neanderthal mtDNAs (Fig. 2 and Dataset S2*A*) confirmed their placement within the variation of Late Pleistocene Neanderthal mtDNA (Fig. 2*A*). The sequences of the Late Neanderthal individuals from both Belgium (Goyet) and France (Saint-Césaire) fall on a phylogenetic branch together with the vast majority of other Late Neanderthals. The mtDNA from Trou Magrite also belongs to this branch, suggesting its temporal association with Late Neanderthals. In fact, with the exception of the mtDNA sequences from Les Cottés Z4-1514 and Grotte Mandrin in France (21, 34), all other Late Neanderthal individuals spanning from Iberia (El Sidrón 1253) to the Caucasus (Mezmaiskaya 2) cluster into subbranches of the same mtDNA lineage.

Specifically, the newly sequenced mtDNA from Goyet 2878-2D is phylogenetically close to one individual from Spy in Belgium and one from Riparo Broion in Italy (Fig. 2). The specimen from Goyet was radiocarbon dated to 37 to 36 ka cal BP (5). However, this date, which postdates the expected time for the demise of Neanderthals ~40 ka (35), is likely affected by contamination with varnish (5). In addition, a pairwise distance analysis shows that the mtDNA sequence of Goyet 2878-2D is identical to that of Goyet Q56-1 (*SI Appendix*, Table S5), an individual from the same archaeological site directly dated to 43 to 42 ka cal BP (5). This suggests a similar date for Goyet 2878-2D, which is within the previously proposed time interval for the entire Goyet Neanderthal assemblage from 45 to 41 ka (5). Similarly, the post-40 ka radiocarbon date obtained here for the newly sequenced specimen Goyet Q305-1 (37,560 to 36,430 cal BP) is probably due to collagen contamination (*SI Appendix*, section 1). In fact, the Goyet Q305-1 mtDNA groups with Goyet Q305-4 and Feldhofer 2, the latter being a Late Neanderthal from the Kleine Feldhofer Grotte in the Neander Valley dated to 44 to 42 ka. Finally, the Trou Magrite 2422-36 individual from Belgium (not directly dated) clusters with other mtDNA sequences from Kleine Feldhofer Grotte (Feldhofer 1), Goyet (Goyet Q57-2), and an individual from Vindija cave in Croatia (Vindija 33.25).

Due to limited DNA preservation and high levels of DNA contamination, specimens Goyet D183-4 (directly dated here to 38,880 to 37,030 cal BP but most likely also artificially younger due to collagen contamination) and Saint-Césaire RPB_117 (stratigraphically dated to ~60 to 55 ka, (36)) were not included in the phylogenetic analysis. However, we were able to perform a tentative placement of these sequences within the Neanderthal mtDNA tree by inspecting the diagnostic substitutions of each subbranch within the whole phylogeny (*SI Appendix*, section 4 and Dataset S2*C*). Goyet D183-4 and Saint-Césaire RPB_117 mtDNAs fall on the Late Neanderthal lineage, consistent with their temporal association (Fig. 2). To the limit of our resolution, Saint-Césaire RPB_117 diverges from the main node of the Late Neanderthal branch whereas Goyet D183-4 branches off from a more derived position of the phylogenetic tree, on a subbranch that includes other individuals from Belgium as well as Germany and Croatia (*SI Appendix*, Figs. S12 and S13). These results highlight close mtDNA affinities between Late Neanderthal individuals across wide geographic distances, linking western and southern European Late Neanderthal populations.

Since the sites of Goyet, Vindija, and Kleine Feldhofer Grotte contain more than one individual with mtDNAs falling on distinct sublineages, we aimed at comparing the level of genetic diversity at each of those sites with what is observed among Late Neanderthals. We computed the pairwise mtDNA distance among sequences within each archaeological site and between all individuals falling on the main Late Neanderthal mtDNA branch (Fig. 2*B* and *SI Appendix*, section 5). The genetic diversity of individuals from Goyet, which is the most sampled site, as well as from Vindija and Feldhofer, is not significantly different from that of the entire main Late Neanderthal branch (Fig. 2*B* and *SI Appendix*, Table S4). This is particularly remarkable because, at least for Goyet, the Neanderthal skeletal assemblage is most likely not representative of a general Neanderthal population but the result of anthropogenic activities, including selective cannibalism (37).

MIS 5-4 Neanderthal specimens from Europe and the Altai (123 to 57 ka) are part of more basal branching lineages on the phylogeny. Within Europe, two individuals dated to ~120 ka from Hohlenstein-Stadel (Germany) and Scladina Cave (Belgium) fall on two distinct branches, which are the most basal of the Late Pleistocene mtDNA tree (11, 20) (Fig. 2*A*). Two of our newly sequenced Neanderthals from Tourtoirac in France (N.1 and N.4), where Neanderthal occupation is indirectly dated to before MIS 3 i.e., older than 57 ka (38), fall on the same branch as Scladina I-4A (*SI Appendix*, Figs. S14–S15). Instead, most of the other MIS 5-4 Neanderthals from Europe fall on a separate branch, which includes two mitochondrial genomes from western Russia (Mezmaiskaya 1 and 3) and one from Poland (Stajnia S5000). Among the newly sequenced individuals, Pešturina 3 from Serbia, which was indirectly dated to 111 ± 11 ka (39), and an additional pre-MIS 3 Neanderthal individual from Tourtoirac (D_14; *SI Appendix*, Fig. S16) are also associated with this branch. The three individuals analyzed from Tourtoirac are believed to belong to the same Neanderthal occupation phase (*SI Appendix*, section 1). Although their mtDNA sequences are incomplete, our SNP analysis indicates that they fall into two distinct mtDNA lineages, suggesting greater genetic diversity at Tourtoirac than observed at single Late Neanderthal sites (Fig. 2*B*). Another Neanderthal mtDNA generated in this study, and associated with the main MIS 5-4 branch, comes from the fetal remains of Sesselfelsgrotte 1 (*SI Appendix*, Fig. S17). While this individual has not been directly dated, thermoluminescence dating of the sediment just above its depositional context yielded ages of 51.1 ± 10.3 ka and 57.5 ± 12.8 ka (40). These stratigraphic estimates, together with its phylogenetic placement, could support a pre-MIS 3 attribution for Sesselfelsgrotte 1. However, future efforts to date the full cave stratigraphy may help clarify the chronological and cultural context of this individual.

In a separate phylogenetic analysis that includes a larger dataset of less well preserved mtDNA sequences (*SI Appendix*, Fig. S19), the same main MIS 5-4 branch also includes three pre-MIS 3 Neanderthals from two sites in the Iberian Peninsula (Estatuas pit II Layer 2, Estatuas pit I Layer 3, and Forbes Quarry). The only Late Neanderthal individual nested within the mtDNA diversity of MIS 5-4 European Neanderthals is the recently published mtDNA from Mandrin Cave in France (Thorin), which was dated to 50 ka (34). Thus, similar to the main Late Neanderthal mtDNA branch, this primarily pre-MIS 3 Neanderthal branch also includes European individuals with a wide geographic distribution, spanning from Iberia to the Caucasus.

In summary, the geographic distribution of Neanderthal mtDNA lineages demonstrates a clear decline in genetic diversity between MIS 5 to 4 and MIS 3 (*SI Appendix*, section 6). Multiple lineages were represented across Eurasia during MIS 5 to 4, while most MIS 3 Neanderthals belong to a single phylogenetic branch, alongside the rare persistence of more deeply divergent mtDNA sequences in southwestern France (*SI Appendix*, Fig. S21). This reduction in lineage diversity supports a major demographic shift in the genetic history of Late Neanderthal populations.

### Molecular Dating and Demographic Analyses.

**Fig. 3.**
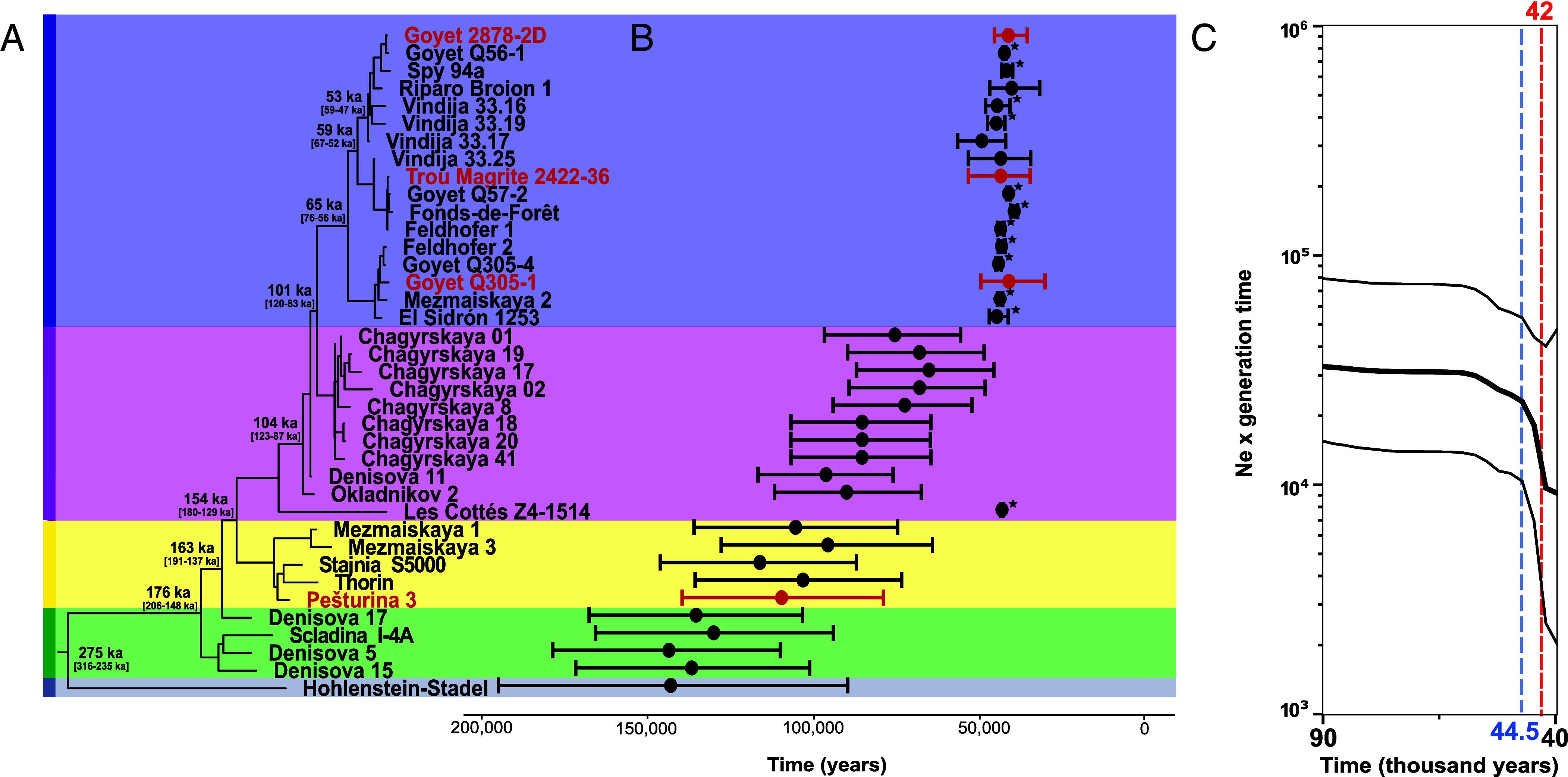
Molecular dating analysis and effective population size estimation as calculated using a Bayesian coalescent skyline approach implemented in BEAST v.2.6.7 (*Materials and Methods*). Four newly generated sequences with more than 93% completeness are included in this analysis and reported in red (*SI Appendix*, Table S8). (*A*) Maximum clade credibility tree of highly preserved mtDNA Neanderthal sequences with the mean and 95% HPD intervals of the divergence dates indicated on major nodes. (*B*) Molecular dating of the mtDNA sequences for nonradiocarbon dated individuals, while the dates of radiocarbon dated individuals used as time anchors are indicated with asterisks. (*C*) Bayesian Skyline plot reporting the change in effective population size (Ne) of western Neanderthals over the time between 90 ka and 40 ka (median value bordered by 95% HPD intervals). The blue and red dashed vertical lines indicate the times for the start of the sharp decline in Ne and the minimum Ne, respectively.

In order to estimate the date of major diversification events within the mtDNA tree and assess the age of nondirectly dated Neanderthal specimens, we performed a molecular dating analysis. For this analysis, we retained Goyet 2878-2D, Goyet Q305-1, Trou Magrite 2422-36, Pešturina 3, and other previously published Neanderthal mtDNAs with low proportions of missing data (*Materials and Methods*). Using a Bayesian statistical framework, we calculated the divergence dates of major nodes within the phylogeny (*Materials and Methods* and Fig. 3*A* and *SI Appendix*, Table S9). The deepest divergence of the Hohlenstein-Stadel lineage is estimated to ~275 ka [95% highest posterior density (HPD) interval: 316 to 235 ka], confirming the date reported in Posth et al. (11). The origins of more derived branches were dated between 177 ka (95% HPD interval: 206 to 148 ka) and 101 ka (95% HPD interval: 123 to 87 ka) and gave rise to most of the pre-MIS 3 Neanderthal mtDNA sequences from the Altai and Europe, including Pešturina 3. Finally, we estimate the diversification of the main Late Neanderthals mtDNA branch to around 65 ka (95% HPD interval: 76 to 56 ka). This coalescence event was preceded by a period of stasis, which lasted over 30,000 y, when no identified mtDNA lineage diversification took place.

Furthermore, we estimated the molecular ages of the newly generated mtDNA sequences where direct dates were unavailable (Trou Magrite 2422-36), likely influenced by contamination (Goyet 2878-2D, Goyet Q305-1), or beyond the radiocarbon limit (Pešturina 3). Specimens Goyet 2878-2D, Goyet Q305-1, and Trou Magrite 2422-36 resulted in an average age of 41 ka (HPD 95%: 46 to 36 ka), 41 ka (HPD 95%: 50 to 30 ka), and 44 ka (HPD 95%: 53 to 35 ka), respectively (Fig. 3*B* and *SI Appendix*, Table S8). These values largely overlap with the Late Neanderthal temporal range and previously published radiocarbon dates for the other individuals from Goyet. In addition, Pešturina 3 is molecularly dated to 110 ka (95% HPD interval: 140 to 79 ka), consistent with the previously obtained OSL and ESR dates to MIS 5e-5c (39). Thus, the results for our newly reported individuals from Belgium and Serbia show congruence between molecular dating and other indirect dating methods based on stratigraphy or associated remains. When extending this analysis to previously published individuals without direct radiocarbon dating, we observe a general pattern of increased antiquity moving more basally along the phylogenetic tree. In fact, all individuals falling on the main Late Neanderthal branch result in a narrow HPD 95% interval (± 7,000 y) with a mean age spanning between 44 ka and 40 ka. Despite diverging from a deeper branch and when running an analysis without fixing its radiocarbon date, the Late Neanderthal individual Les Cottés Z4-1514 provides a molecular date of 64 ka (HPD 95%: 91 to 41 ka). While the mean molecular date is older than its direct date (43,740 to 42,720 cal BP), the HPD interval overlaps (*SI Appendix*, Fig. S18). Instead, all individuals belonging to the main MIS 5-4 Neanderthal European branch, including Pešturina 3, Mezmaiskaya 1 and 3, Stajnia S5000 and Thorin, provide an average molecular date around 100 ka despite a much larger HPD 95% interval (± 25,000 y). These dates are largely consistent or marginally older than those estimated for each individual through other dating techniques, with the exception of Thorin, which has been dated twice as young as the molecular dating inferred (34). When we fix Thorin to 50 ka, all individuals on this branch become younger but do not reach an estimated mean date consistent with that of Late Neanderthals (*SI Appendix*, Fig. S18). Finally, the two European individuals falling on more basal lineages, Hohlenstein-Stadel and Scladina I-4A, result in an age around 120 ka, in line with previously published estimations (11, 20).

In order to assess the robustness of the Bayesian analysis to molecularly date specimens, we implemented a tip-to-root regression analysis using TempEst (41) on a previously computed Maximum Parsimony tree. Using either the modern human reference sequence (revised Cambridge Reference Sequence, rCRS) or the Hohlenstein-Stadel sequence to root the tree, we observe a general trend where phylogenetically more derived sequences have greater distance from the tree root compared to more basal sequences, consistent with the dating results obtained with BEAST2 (Fig. 3*B* and Dataset S2*B*). However, there are two notable exceptions. The first one is the Les Cottés Z4-1514 mtDNA which, despite branching off more basally than the main Late Neanderthal branch, has accumulated the greatest number of substitutions among all Neanderthals. The second is Thorin, which instead shows a limited number of substitutions similar to pre-MIS 3 Neanderthals. Since there might be substantial rate variation among tree branches, we cannot establish if the short branch length observed for Thorin is due to an older date, a faster mutation rate along its mtDNA lineage, or both. In fact, contrary to the main Late Neanderthal branch, we are currently limited in our ability to calibrate deep events in the Neanderthal mtDNA tree by the scarcity of radiocarbon dated individuals falling on more deeply divergent branches. Once reliable direct dates for individuals with mtDNA diverging from the primarily MIS 5-4 Neanderthal branches become available as calibration points, it will be possible to perform more reliable and accurate molecular dating estimates for older events in the evolutionary history of Neanderthals.

Finally, we calculated changes in effective population size in the maternal lineages of western Neanderthals using a Coalescent Bayesian Skyline analysis (Fig. 3*C*). While this analysis does not have resolution to detect expansion and/or contraction in pre-MIS 3 Neanderthal history likely because of the sparse sampling of a single genetic locus (35), we observed a demographic change during the Late Neanderthal phase, the period with the largest amount of available genetic data. Specifically, our analysis identifies a rapid decline in the effective population size of Late Neanderthals around 44.5 ka, which reaches a minimum at ~42 ka, soon before their disappearance (42).

### Temporal Changes in Archaeological Assemblages.

**Fig. 4.**
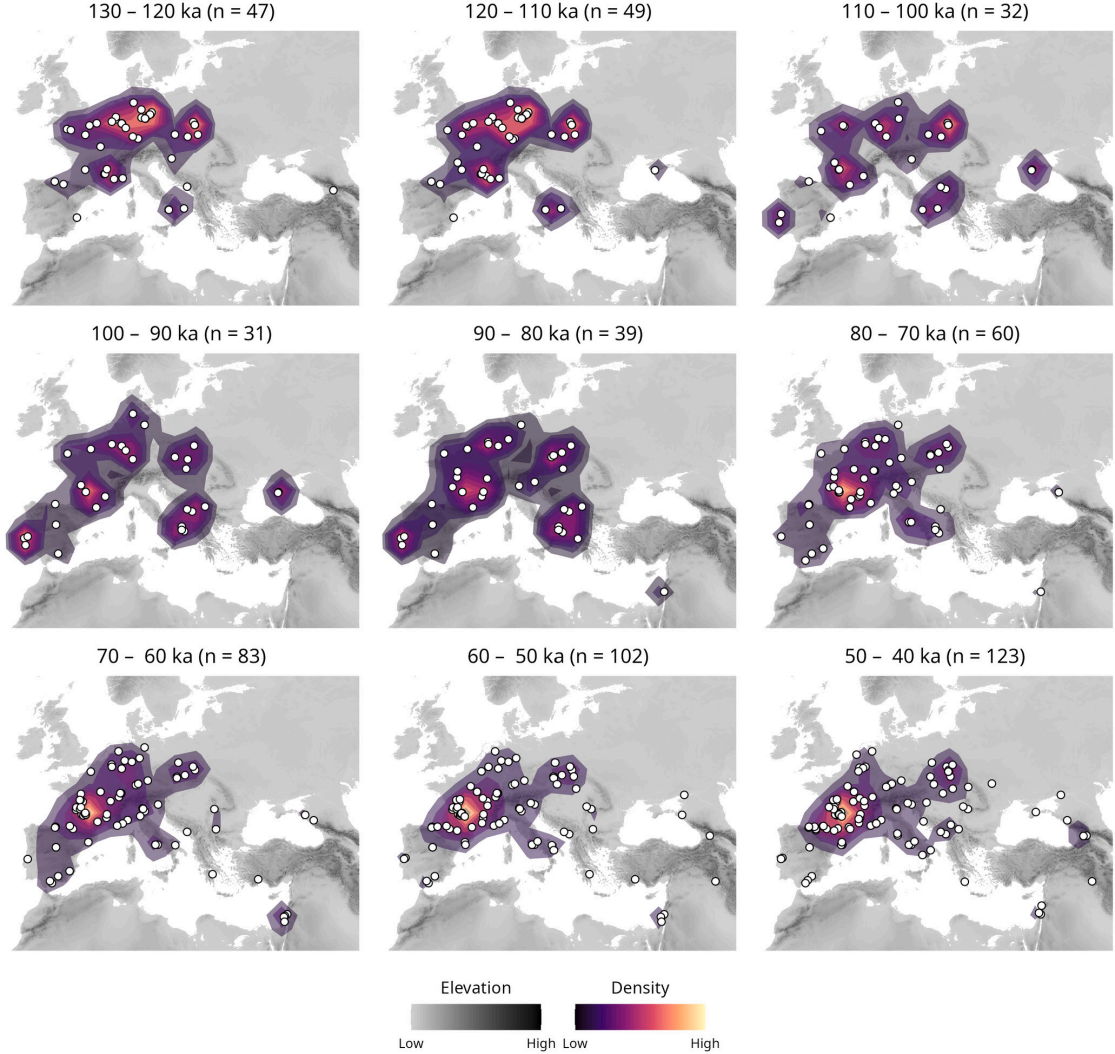
Geographic distribution of archaeological sites (n) in western Eurasia associated with Neanderthals between 130 and 40 ka in 10,000-y sliding windows (Neanderthal sites outside western Eurasia have been cropped out of the maps to facilitate visibility). Each map reports elevation on a gray scale and density of archaeological sites in color, as indicated in the legend.

To contextualize the genetic results obtained in this study within an archaeological framework, we investigated the distribution of Neanderthal archaeological assemblages from 130 ka onward leveraging on the ROCEEH Out of Africa Database ROAD dataset (43). We acknowledge that the spatial and temporal presence for archaeological evidence associated with Neanderthals is likely influenced by multiple biases such as research history, dataset collection and preservation, as well as geological constraints. Nevertheless, we aimed at capturing different facets of Neanderthal archaeological presence by conducting three separate queries of ROAD: 1) all available European Middle Paleolithic sites dating prior to the arrival of modern humans in Europe, 2) all available sites containing Neanderthal skeletal remains and 3) all available sites associated with technocomplexes that are traditionally linked to Neanderthals (*Materials and Methods* and *SI Appendix*, section 7). These data were then filtered, keeping only assemblages with up to 30,000 y range as entered in ROAD. Through time-slices of 10,000 y, we plotted the recorded presence of Neanderthals across western Eurasia (Fig. 4). In this analysis it becomes apparent that from 130 ka to 80 ka the distribution of archaeological evidence in Europe associated with Neanderthals fluctuates, and is concentrated in multiple areas spanning from the Iberian Peninsula on the west to the Black Sea on the east. Then, from 80 ka onward, a contraction in the distribution of archaeological assemblages is observed with the highest density found in southern France between 70 ka and 60 ka, potentially indicating a demographic and/or cultural refugium during a period of population contraction. Subsequently, while the density hotspot remains stable, a larger number of individual sites are distributed across most of western Eurasia, suggesting a phase of geographic re-expansion. This pattern may reflect shifting adaptive strategies in response to changing climatic and ecological conditions, as well as the complex demographic dynamics of Late Neanderthal populations across western Eurasia.

To evaluate the robustness of spatial patterns in the ROAD dataset, we applied the local Getis–Ord Gi* statistic, which identifies statistically significant clusters (“hotspots”) of Neanderthal site density while accounting for spatial dependence and uneven sampling. This approach complements the kernel density visualizations by providing a formal spatial test of clustering (*SI Appendix*, section 9). The results show a contraction of hotspot area from 130 to 110 ka to 90 to 80 ka, followed by renewed and intensified clustering after ~80 ka (*SI Appendix*, Table S10). From 80 to 70 ka onward, emerging and persisting hotspots are concentrated in western Europe, while hotspots in other regions tend to disappear (*SI Appendix*, Figs. S22 and S23). By 60 to 50 ka, statistically significant concentrations appear in France, consistent with a southwestern European focus of Neanderthal activity during the final phases of their existence (*SI Appendix*, Table S11). Structured permutation tests demonstrate that this pattern cannot be explained by random spatial sampling alone, supporting the interpretation that it reflects genuine demographic persistence rather than discovery bias (*SI Appendix*, Tables S11 and S12).

We furthermore used a locality-level rarefaction analysis to test whether the apparent late-stage geographic broadening could be explained by larger sample sizes. Using 80 to 70 ka as the baseline, we repeatedly downsampled younger slices to the same number of localities (n = 60; 1,000 replicates) and quantified longitudinal spread via longitude range (full and central 95%) and occupancy of 2° longitude bins. After rarefaction, longitudinal spread increased progressively: 70 to 60 ka showed a significantly broader central 95% range than 80 to 70 ka (40.0° vs 32.3°, *P* = 0.009), and both 60 to 50 ka and 50 to 40 ka showed significantly larger full and central ranges (full: 51.9° vs 47.2°, *P* = 0.045 and 0.022; central 95%: 43.5° and 47.0°, *P* = 0.011 and 0.004). All post-80-70 ka slices also occupied additional longitude bins absent from the baseline (median 2 to 3 additional bins), supporting a real post-80-70 ka widening of geographic distribution that is not attributable to increased sample size alone (*SI Appendix*, Table S13).

Additionally, we replicated the analysis conducted by Yaworsky and colleagues (44) to investigate the fundamental climatic niche space of Neanderthals from 130 to 40 ka. In recent years, the ROCEEH team has concentrated on integrating more European data into ROAD, thereby enabling the application of the same approach to a broader dataset. Our analysis combines paleoclimate reconstructions, a spatiotemporal species distribution model and dated contexts from Neanderthal sites across western Eurasia using the current version of ROAD (*Materials* *and Methods* and *SI Appendix*, Fig. S25 and section 8). We confirm a general decline in the size of the niche space of Neanderthals from the interglacial stage MIS 5e onward, with the lowest projected potential niche space—excluding the time of the Neanderthal disappearance—at around 65 ka, within the peak of MIS 4, a glacial period marked by cold and dry climatic conditions (45).

Together, these findings underscore the close relationship between environmental change and Neanderthal spatial organization, both in terms of archaeological site distribution and ecological niche modeling.

## Discussion

Our study expands the archaic human mtDNA dataset, illuminating on the genetic processes that accompanied the last phases of Neanderthal evolution leading to their extinction. Following targeted enrichment and next generation sequencing, we assembled ten complete or partial Neanderthal mitochondrial genomes associated with Late Neanderthals from both MIS 5-4 and MIS 3. These include three additional mtDNA sequences from Goyet (Belgium), three mtDNA sequences from the Tourtoirac rock shelter (France), as well as one from each of the following archaeological sites: Pešturina (Serbia), Saint-Césaire (France), Trou Magrite (Belgium), and Sesselfelsgrotte (Germany). The data from Trou Magrite increase the total number of sites in Belgium with identified Neanderthal specimens to nine (24). We coanalyzed the newly generated sequences with previously published mtDNA sequences spanning western Eurasia and the Altai in southern Siberia.

The mtDNA phylogenetic analyses show a strong temporal signal and the lack of a phylogeographic pattern in Late Neanderthals. In fact, all but two of the 22 currently available Late Neanderthal mitochondrial genomes cluster within a single lineage, which includes individuals spanning a large portion of Europe, from Spain to the Caucasus. In contrast, older European Neanderthals, including the newly reported pre-MIS 3 Neanderthal mtDNAs from Pešturina and Tourtoirac, as well as the one from Sesselfelsgrotte, are placed on more deeply divergent branches and fall outside the genetic variation of Late Neanderthals. This pattern is particularly evident when we examine the mtDNA diversity at a single site such as Goyet, which, in a maximum time frame of ~4,000 years (45 to 41 ka), encompasses nearly the entire diversity observed in Late Neanderthals across Europe. Thus, Neanderthal mtDNA diversity declined from MIS 5 to 4 to MIS 3, with a single Late Neanderthal lineage widely distributed across Europe, indicating a major demographic shift. These findings suggest that Late Neanderthals emerged from a population bottleneck or from a continuous small effective population size that led to the loss of most of the earlier mtDNA lineages. Interestingly, a recent morphological analysis of Neanderthal semicircular canals also points to a similar bottleneck event (46). In addition, European Neanderthals from MIS 5-4 seem to have followed a similar evolutionary trajectory, with their mtDNA clustering mainly on two mtDNA branches irrespective of geography. Taken together, these results suggest that there were recurring patterns of geographic contraction and expansion throughout Neanderthal population history, which were possibly driven by major climatic shifts during the last Ice Age (47).

Using a Bayesian statistical framework, we estimate the molecular age of the newly generated mtDNA sequences, showing congruence with indirect dating methods based on stratigraphy or associated remains. These molecular dating results contribute further evidence to challenge the direct radiocarbon dates for the Goyet specimens that are younger than 40 ka, including two dates obtained in this study. In addition, they provide an MIS 3 molecular date for the nonradiocarbon-dated neonate femur from Trou Magrite. Furthermore, we estimate that the emergence of the main Late Neanderthal mtDNA branch took place roughly 65 ka and was preceded by a period of stasis when no mtDNA diversification events occurred. This period, which lasted over 30,000 y, spanned MIS 4, a glacial stage characterized by harsh climatic conditions (45). In particular, a glacial maximum between 73 and 60 ka (48), likely confronted European Neanderthals with considerably deteriorating climatic conditions. Thus, our results are consistent with climate-driven population turnover that occurred toward the end of Neanderthal history from which the vast majority of Late Neanderthal mtDNA lineages derive.

In addition to dating the origins of the Late Neanderthal mtDNA branch, we attempted to infer possible locations of glacial refugia during MIS 4 that gave rise to most Late Neanderthal mtDNA diversity. Drawing on almost 20 y of data collection in ROAD, we tracked the geographic shifts of archaeological assemblages over the course of 90,000 y of Neanderthal history (130 to 40 ka). The combination of genetic and archaeological data shows that Late Neanderthals followed a large-scale population replacement, possibly originating from a geographically confined group in southwestern France before their redistribution across other areas of Europe. This corroborates the hypothesis of a major ecological niche shift in western Europe around 70 ka which appears to have led Neanderthals to develop novel technological solutions and adopt different mobility patterns, enabling them to inhabit the same territories as during MIS 5 (49). However, we argue that this ecological niche contraction may have had continental-level effects, leading to high genetic homogeneity among Late Neanderthals in Europe after ~65 ka.

This event largely replaced older Neanderthal lineages with a single, rapidly expanding mtDNA lineage as evidenced by the near-absence of surviving pre-65 ka mtDNA diversity among Late Neanderthals. Notable exceptions include Thorin and Les Cottés Z4-1514 in France, whose mtDNA lineages persisted after the turnover event, suggesting the preservation of higher genetic diversity in the southwestern European glacial refugium. Recognizing possible biases in the genetic and archaeological records, the genetic findings presented here align with archaeological evidence from ROAD, which indicates a reduction in the accumulation of Neanderthal assemblages around the time of the inferred genetic bottleneck and a re-expansion thereafter. While the genetic data show a high degree of homogeneity among Late Neanderthal populations, the material culture of this period is far from homogeneous. Future paleogenetic research may ultimately be able to provide insights into the complexity of the process of cultural diversification and isolation that likely preceded Neanderthal extinction.

Finally, we identify a rapid decline in the effective population size of Late Neanderthals after ~45 ka, reaching a minimum around ~42 ka, possibly corresponding to the period of their final disappearance. This result corroborates the analysis of the potential Neanderthal niche space across western Eurasia, which reached a minimum at the end of the Neanderthal’s existence (44).

In conclusion, our study reveals a complex demographic history for Late Neanderthals, marked by a population contraction followed by re-expansion, ultimately leading to an almost complete replacement of mtDNA lineages across Europe. Although only the generation of additional Neanderthal nuclear genomes will determine the extent of this population turnover, the integration of mitochondrial genomes with archaeological evidence offers crucial insights into the timing and geographic context of the events that shaped the genetic makeup of Late Neanderthals.

## Materials and Methods

### Archaeological Materials.

Ten samples (Fig. 1 and *SI Appendix*, Table S1) were obtained from six archaeological sites. The material from Goyet and Trou Magrite is housed at the Royal Belgian Institute of Natural Sciences in Brussels. The Saint-Césaire RPB_117 specimen comes from the excavations of F. Lévêque at La Roche-à-Pierrot in Saint-Césaire (50) and is housed at the Musée d’Archéologie nationale in Saint-Germain-en-Laye, France. The Tourtoirac N. 1 and N. 4 samples come from H. Laville’s excavations at the site (1968–72) and are curated at the Musée national de Préhistoire in Les Eyzies, France. Tourtoirac D_14 is temporarily housed at the CNRS UMR5199 PACEA laboratory, University of Bordeaux, France, and comes from the ongoing excavations of L. Doyon at the site. The Sesselfelsgrotte 1 fetus is housed at the Institute for Pre- and Protohistory, Friedrich-Alexander-Universität Erlangen-Nürnberg, Germany. The Pešturina 3 molar is housed at the Department of Archaeology of the University of Belgrade, Serbia. Permits to sample the material for radiocarbon dating and genetic analyses were provided by the respective institutions.

### Laboratory Procedures.

Sampling was performed at the clean room facilities of the Royal Belgian Institute of Natural Sciences in Brussels, Belgium (Goyet D183-4, Trou Magrite 2422-36), the University of Tübingen, Germany (Goyet Q305-1, Goyet 2878-2D, Sesselfelsgrotte 1, and Tourtoirac N. 1, N. 4, D_14), and the Max Planck Institute for the Science of Human History in Jena, Germany (Saint-Césaire RPB_117, Pešturina 3). A dentist drill was used in each case to sample bone powder, weighing between 8.4 and 77 mg. DNA extraction was carried out at the University of Tübingen and Max Planck Institute for the Science of Human History following an established protocol (28). 30 µL of each extract was used to build single stranded, double indexed, non-UDG and UDG-half-treated libraries (29). Negative and positive controls were taken throughout the entire workflow. Libraries were then enriched for the entire mitochondrial DNA via in-solution capture (31, 51) and sequenced on an Illumina HiSeq4000 (1x75 cycles) for up to ~70 M reads per sample (Dataset S1).

### MtDNA Mapping and Consensus Reconstruction.

The sequencing data from each run were merged and subsequently run through the EAGER pipeline (52). CircularMapper (parameters –n 0.01, –l 16500, –q 30) was used to align the merged sequences to the RNRS (Reconstructed Neanderthal Reference Sequence). DeDup (52) was used to remove duplicates and mapDamage2 (53) to determine damage patterns. The resulting alignment (BAM) files were converted to FASTQ format and then mapped against the rCRS (revised Cambridge Reference Sequence) in EAGER using CircularMapper with lenient parameters (–n 0.0001, –l 16500, -q 30) to be able to run schmutzi (32). Schmutzi was implemented to calculate contamination estimates and to create a consensus FASTA file with quality filter q30 (*SI Appendix*, Table S2). For the samples with less than 10% contamination (Goyet Q305-1 and Trou Magrite 2422-36) the resulting consensus sequence was used for later analyses. For the other samples, in order to reduce the effect of modern human contamination on consensus calling, we filtered the reads based on the presence of C-to-T mismatches at the first and/or last three positions (deaminated fragments) (33). We then lowered the base quality of the deaminated positions and trimmed the ones showing a base quality of less than 20 for up to three positions from both ends. For each position a base was called when covered by at least three independent DNA fragments and 65% of the fragments carried the same base using Geneious Prime v.2022.1.1.

### Database Construction.

Neanderthal and Denisovan mitochondrial genomes were collected using two complementary approaches: 1) NCBI BLAST searches and 2) manual curation from the literature. For the BLAST search, we used the reconstructed Neanderthal reference sequence (RNRS) as a query against the core nucleotide database, restricted to the taxonomic group *Homo sapiens neanderthalensis* (taxid:63221), and optimized for highly similar sequences using MegaBLAST. Manual curation involved reviewing relevant publications and associated supplementary materials. In total, we identified 63 Neanderthals and 17 Denisovans with more than 45% complete mtDNA sequences (Dataset S2*A*). To minimize redundancy and individual overrepresentation, we excluded duplicate sequences. Individuals within an archaeological site were considered duplicates if they showed no variable sites across their mitochondrial genomes, acknowledging that while identical mtDNA can be shared among maternally related individuals or even within small populations, this conservative filtering was necessary to reduce potential biases in downstream analyses. Two sites were considered for duplicate pruning: 1) Chagyrskaya, from which 14 out of the 18 published mtDNA sequences belong to different individuals (54); 2) Goyet, from which three out of the seven reported mtDNA sequences belong to different individuals (55). Furthermore, sequences with high levels of missing data—defined as mtDNAs with more than 2,600 unresolved nucleotide positions (less than 85% complete)—were excluded. This threshold was established based on experimental construction of Maximum Parsimony trees, where we observed that sequences exceeding this level of missing data caused instability in phylogenetic topology and led to biologically implausible placements. After the application of all filtering steps, our final dataset comprises 49 Neanderthal and 9 Denisovan published mtDNA sequences (Dataset S2*A*).

### Phylogenetic Analysis.

In order to investigate the maternal relationships of our newly generated sequences with previously published Neanderthal mtDNAs, we followed two different approaches, one based on Maximum Parsimony and the other based on single nucleotide polymorphisms (SNPs) (*SI Appendix*, section 4). For the Maximum Parsimony analysis, we built two multiple genome alignments using MUSCLE (56). The first includes the mtDNA of the full list of 49 previously published and four newly assembled Neanderthals (Goyet Q305-1, Goyet 2878-2D, Pešturina 3, and Trou Magrite 2422-36), one Sima de los Huesos, 9 Denisovans, 55 modern humans, and *Pan troglodytes* as an outgroup (total 119 mtDNAs) all displaying a maximum of 2,600 Ns (85% completeness). With this alignment a Maximum Parsimony tree was built using MEGA X (57) with a 90% partial deletion over the entire mtDNA (16,529 considered positions) and 500 bootstrap iterations (Dataset S2*A* and *SI Appendix*, Fig. S19). In the second multiple genome alignment, we combined 34 mtDNAs from previously published Neanderthal individuals and four Neanderthal mtDNAs assembled in this study with a maximum of 104 Ns (99.4% complete), and rCRS as an outgroup (total 39 mtDNAs). A Maximum Parsimony tree considering the entire mtDNA was then built with complete deletion (16,275 considered positions) and 500 bootstrap iterations (Dataset S2*A* and *SI Appendix*, Fig. S20). Finally, the same alignment was trimmed off the D-loop to generate a coding-region-only Maximum Parsimony tree with complete deletion (15,359 considered positions) and 500 bootstrap iterations (Fig. 2). For the other six individuals (Saint-Césaire RPB_117, Goyet D183-4, Sesselfelsgrotte 1, Tourtoirac N.1, N.4 and D_14) with a higher level of data missingness (more than 1,100 Ns) we refrained from computing them into a Maximum Parsimony tree. Instead, we performed a tentative phylogenetic placement by inspecting the presence or absence of derived alleles along the Neanderthal mtDNA phylogeny. First, we identified the SNPs that define every node (*SI Appendix*, Fig. S11 and Table S3) and evaluated the allele frequency at each SNP after omitting sites at which base calls could be the result of deamination. To highlight their uncertain phylogenetic position, these samples are included in the reported trees with red dashed lines (Fig. 2 and *SI Appendix*, section 4).

### Bayesian Analyses.

The software package BEAST2 v. 2.6.7 was used to estimate the divergence times between and within hominin lineages, as well as the molecular date of undated Neanderthals. For this analysis, we considered the same well-preserved published and newly generated Neanderthal mtDNAs as in Fig. 2, plus 55 modern human mtDNAs and the Denisova 3 mtDNA as an outgroup. We created a multiple genome alignment with only the coding region, and all sites with missing/ambiguous data and gaps were eliminated, resulting in 15,350 positions. To find the best substitution model, we ran the fasta alignment in Modelgenerator v. 85 (58) and bModelTest (59), both identifying Tamura-Nei 93 with invariable sites as the best-fitting model (60). As calibration points, we used the radiocarbon dates from directly dated Neanderthals (*SI Appendix*, Table S7), while we provided a range between 30 and 300 ka (initial value of 165 ka) for the rest of the Neanderthal individuals, and kept to zero the date of the modern human sequences. We tested two tree priors (Coalescent Bayesian Skyline (CBS) and Coalescent Constant Population (CCP)), with a marginal like estimation (MLE) analysis using stepping-stone (SS) sampling from the BEAST2 model-selection package with alpha set to 0.3 and a pre-burn-in percentage of 10%. For each tree prior 100 steps with a chain length of 15,000,000 were run in order to ensure convergence of the majority of the steps. Following Kass and Raftery (61), the model with CBS was favored over CCP (log10 BF > 30). We thus ran BEAST with a CBS tree prior and two clock rates (strict clock or log normal-distributed relaxed clock). For consistency with previous studies (i.e., (11, 21)), we describe in the main text the results from the CBS with strict clock and a substitution rate of 1.57 × 10^−8^ substitutions/bp/year (30) and report the results from the CBS with log normal-distributed relaxed clock in the *SI Appendix*, section 6. Both analyses were run four times with a chain length of 200,000,000 iterations, and a sampling frequency of 10,000 for the MCMC. We discarded 10% of the states from each run as chain burn-in and then combined the four independent runs using the LogCombiner v1.8.4 resulting in 135 million iterations. A clear shift from the prior to the posterior distribution was observed, indicating that the selected priors were not overly constraining (*SI Appendix*, Table S6). A second CBS analysis was performed in BEAST2 to specifically track changes in maternal Effective Population Size (Ne) of western Eurasian Neanderthals over time. We created a multiple genome alignment with the mtDNA coding region of 21 published and four newly generated western Eurasian Neanderthal individuals (i.e., excluding published Neanderthals from the Altai region). All sites with missing/ambiguous data and gaps were eliminated resulting in 15,446 positions. The best-fitting substitution model was again Tamura-Nei 93 with invariable sites, as determined by both Modelgenerator and bModelTest. We included the same calibration points as in the previous molecular dating analyses. However, since we did not include any present-day individual in this CBS analysis, we considered as the zero date the Neanderthal individual with the youngest mean radiocarbon date in our dataset (Fonds-de-Forêt 1, 39.5 ka) and adjusted all other calibration points accordingly (subtracting 39,500 y). The molecular clock was set to strict with a substitution rate of 1.57 × 10^−8^ substitutions/bp/year (30). All other parameters were specified identically as in the previous BEAST runs.

### Temporal Changes in Neanderthal Archaeological Assemblages and Climatic Niche Space.

To extract relevant Neanderthal-associated data from the extensive and curated dataset collected in the ROCEEH (The Role of Culture in Early Expansions of Humans) Out of Africa Database (ROAD), we used the publicly available query tool AskROAD (https://www.roceeh.uni-tuebingen.de/askROAD/). We applied three complementary queries targeting different aspects of Neanderthal presence. The first selected European Middle Paleolithic assemblages dated to between 130 and 60 ka. The second targeted assemblages across Europe and Asia with Neanderthal skeletal remains dated from 130 to 40 ka. The third query focused on technocomplexes within the same timeframe associated with Neanderthals (Mousterian, Micoquian) or whose association is still debated (Châtelperronian). Query results were downloaded as CSV files and processed in R. We combined, filtered, and cleaned the datasets, retaining only those with date ranges up to 30,000 y. The data were then divided into 10,000-y time slices to assess temporal changes. Kernel Density Estimation (KDE) was used to generate heatmaps showing the spatial intensity of Neanderthal sites. KDE increases density values in regions where data cluster, producing “hotspots” on the maps (Fig. 4). We reproduced the analysis by Yaworsky and colleagues (44), which examines Neanderthal climatic niche space, and confirmed their results. The code is openly available on Zenodo, and the data were drawn from ROAD on July 30th 2025 via dynamic PHP scripts that update as additional entries are added. The total number of archaeological sites included in the original study (n = 94) is increased in our reanalysis (n = 131, *SI Appendix*, Fig. S24).

## Supplementary Material

Appendix 01 (PDF)

Dataset S01 (XLSX)

Dataset S02 (XLSX)

## Data Availability

The raw sequencing files and the mitochondrial DNA sequences of the analyzed individuals in this study are available at the European Nucleotide Archive (ENA) under study accession number PRJEB90662. The four nearly complete mtDNA sequences reconstructed in this work are deposited in GenBank (accession numbers PZ099884, PZ099885, PZ099886, PZ099887). All codes associated with this study as well as the ROAD dataset analyzed here are available at the accompanying research compendium on Zenodo (62). All study data are included in the article and/or supporting information.
