# Supplementary material for: Archaeogenetic insights into the demographic history of Late Neanderthals
Source: Proc Natl Acad Sci U S A. 2026 Mar 23;123(13):e2520565123. doi: 10.1073/pnas.2520565123 (PMC13037871; doi:10.1073/pnas.2520565123)
Supplement: Supplementary file 1 — Appendix 01 (PDF) [file pnas.2520565123.sapp.pdf]

Supporting Information for
Archaeogenetic insights into the demographic history of Late Neanderthals

Charoula M. Fotiadou[1,2], Jesper Borre Pedersen[3], Hélène Rougier[4], Mirjana Roksandic[5], Maria A. Spyrou[1,2], Kathrin Nägele[6], Ella Reiter[1], Hervé Bocherens[2,7], Andrew W. Kandel[3], Miriam N. Haidle[3,8], Timo P. Streicher[3], Nicholas J. Conard[2,8], Flora Schilt[9,10], Ricardo Miguel Godinho[10], Thorsten Uthmeier[11], Luc Doyon[12], Patrick Semal[13], Johannes Krause[1,6], Alvise Barbieri[10], Dušan Mihailović[14], Isabelle Crevecoeur[12], Cosimo Posth[1,2,*]

* Correspondence: cosimo.posth@uni-tuebingen.de

This PDF file includes:

Supporting text
Figures S1 to S25
Tables S1 to S13
SI References

Other supporting materials for this manuscript include the following:

Datasets S1 and S2A,2B,2C

Table of contents

Supporting Information Text 3
1. Archaeological Information 3
1.1 Belgium 3
1.1.1 Goyet (Troisième caverne) 3
1.1.2 Trou Magrite 3
1.2 France 4
1.2.1 Saint-Césaire 4
1.2.2 Tourtoirac rock shelter 4
1.3 Serbia 5
1.3.1 Pešturina Cave 5
1.4 Germany 5
1.4.1 Sesselfelsgrotte 5
2. Extraction and library preparation 6
3. MtDNA mapping and consensus reconstruction 6
4. SNP analysis 6
5. Pairwise nucleotide differences 7
6. Phylotemporal distribution 8
7. BEAST analyses 8
7.1 Quantitative comparison of prior and posterior distributions 8
7.2 Impact of tip dating on molecular age estimates 9
7.3 Effect of clock model on undated specimens 9
7.4 Tip-to-Root analysis 9
7.5 Integrative dating approaches 9
8. Distribution map generation from ROAD data 10
9. Getis–Ord Gi* statistics 12
9.1 Methods — Spatial statistics and rationale 12
9.2 Results — Temporal and spatial trends 12
9.3 Summary on statistical inference and robustness 13
10. Subsampling test of geographic spread 13
11. Reproducing the analysis of Yaworsky et al. 2024 14
Supplementary Figures 15
Supplementary Tables 40
Datasets 53
SI References 54

Supporting Information Text

1. Archaeological Information

1.1 Belgium

1.1.1 Goyet (Troisième caverne)

Belgium has indisputably contributed to the development of paleoanthropology and the acceptance of Neanderthals as a different human taxon from our own (1). Four Belgian cave sites have so far yielded ancient DNA and contributed to the growing paleogenetic sample. The largest contribution comes from the Troisième caverne (or Third Cave) of Goyet, a major Paleolithic site since E. Dupont's excavations in 1868. Although E. Dupont was a geologist with a keen interest in stratigraphy (2), field methods at the time did not meet today's standards and the different levels he identified actually correspond to a mix of material from different chrono-cultural contexts (e.g. 3). In 2008, an interdisciplinary reassessment of the faunal and human collections from Goyet was initiated. Combining the results of morphometrics, taphonomy, stable isotope, dating, and genetic analyses, this project has resulted in the identification of both Neanderthal and Upper Paleolithic human remains (4). The Goyet Neanderthal collection is now the largest assemblage of Neanderthal remains in Northern Europe and several of the specimens have been directly radiocarbon dated (5). They provide a range between 41,000 and 45,000 years cal BP when calibrated with the IntCal20 calibration curve (6).

Ten Neanderthal specimens were initially processed for genetic testing, all preserving evidence of endogenous mtDNA (5). In this study, three previously unpublished mtDNAs from three different Neanderthal individuals are presented (Goyet Q305-1, Goyet 2878-2D and Goyet D183-4).

Goyet Q305-1 (Fig. S1) is a right neonate femur. It was discovered among the material in drawer Q305, expected to contain indeterminate fauna. Goyet Q305-1 showed morphological features pointing to a potential archaic status and was selected for dating and paleogenetic analyses. For radiocarbon dating, collagen extraction was performed at the University of Tübingen following Krajcarz et al. (7, 8). Collagen quality (%Ccoll = 38.7, %Ncoll = 14.0, Ccoll:Ncoll = 3.2) was checked against standard evaluation criteria (9, 10) before being sent to the Centre for Isotope Research of the University of Groningen. Goyet Q305-1 yielded an age of 32,650 ± 210 14C BP (GrA-62471), i.e. 37,560 - 36,430 years cal BP (95 % probability). This age appears too young and is probably due to contamination by recent carbon, a possibility raised by the early developmental stage of the bone resulting in its very porous structure.

Goyet D183-4 is a fragmentary juvenile left clavicle (Fig. S2) also discovered among the fauna from the site. However, it was identified on a tray of well-preserved cave bear remains that used to be displayed in the exhibit of the Royal Belgian Institute of Natural Sciences. As such, all the remains on the tray had been varnished, including Goyet D183-4. The specimen was nevertheless selected for dating and paleogenetic analysis to clarify its status. Collagen extraction, quality evaluation (%Ccoll = 40.9, %Ncoll = 14.6, Ccoll:Ncoll = 3.3), and AMS dating followed the same protocol as for Goyet Q305-1 (see above). With a radiocarbon date of 33,150 ± 220 14C BP (GrM-13123), i.e. 33,880 - 37,030 years cal BP (95 % probability), the age of the Neanderthal clavicle Goyet D183-4 appears too young and can be explained by contamination due to varnish.

Finally, the previously published Neanderthal lower left second premolar Goyet 2878-2D (Fig. S3), which refits into mandible Goyet 2878-8, represents an adult or adolescent individual (5). It was radiocarbon dated yielding ages between 36,970 and 36,160 years cal BP (recalibrated here with IntCal20; 6), which appear too young and can be explained by the presence of unnoticed varnish (5).

1.1.2 Trou Magrite

Trou Magrite is a cave site located at Pont-à-Lesse in the Lesse Valley, commune of Dinant, Belgium. Initially excavated by E. Dupont in 1867 (11), the most recent excavations at the site were conducted by L. Straus and M. Otte in 1991-92 (12). The archaeological record includes an important Middle Paleolithic collection that probably represents several occupation phases as the 1991-92 fieldwork uncovered deposits yielding Mousterian materials that may correspond to MIS 5 to 3 (12). Unfortunately, correlations with E. Dupont's work are impossible in part because the materials from the different "fauna-bearing levels" that he defined in the field were mixed post-excavation (13). E. Dupont discovered several human remains and identified them as Paleolithic humans but they have been only partially published thus far (14). In 2015, an interdisciplinary reassessment of E. Dupont's faunal and human collections was started in the same way as what had been previously done for Goyet (see above). This resulted in the identification of two Neanderthal specimens that were selected for genetic analyses, an upper right permanent canine (TM 2419-10) and a neonate left femur diaphysis (TM 2422-36, Fig. S4). Both had been misidentified as faunal remains. The femur yielded evidence of ancient DNA and was selected for further genetic analyses.

1.2 France

1.2.1 Saint-Césaire

The archaeological site of La Roche-à-Pierrot, a collapsed rock shelter at the base of a limestone cliff, is located in Saint-Césaire, in the Charente-Maritime department of France. The site was accidentally discovered in 1976 during earthworks, after which excavation campaigns were conducted by F. Lévêque until 1987 (15).

La Roche-à-Pierrot is well known for preserving a ~30,000-long sedimentary record of the Middle-to-Upper Paleolithic transition in southwestern France. Archaeological discussions surrounding the site have largely centered on the Mousterian/Châtelperronian layer and the underlying Mousterian bone bed, the latter representing the uppermost horizon of an unequally documented Discoid-Denticulate Mousterian sequence.

In 2001, seven perinatal human remains were identified within the faunal assemblage at the base of the Mousterian layers (16). A subsequent comprehensive reassessment of the Mousterian faunal collections from François Lévêque's 1977–1987 excavations led to the identification of additional perinatal remains, including the left petrous bone RPB_117 (RPB84_E4IV_EGF_30-RPB117) analyzed in the present study (Fig. S5).

Since 2013, systematic excavations at La Roche-à-Pierrot, directed by I. Crevecoeur, have revisited the site's stratigraphy, chronology, and the context of the previously discovered human remains. Multiscalar and multiproxy geoarchaeological analyses have provided new insights into the site's integrity, formation history, depositional processes, and the chrono-cultural framework of the Mousterian occupations (17). Specimen Saint-Césaire RPB_117 derives from the EGC level of Lévêque's excavation, corresponding to Stratigraphic Unit 20 of the new excavations, which is associated with the Discoid-Denticulate Mousterian and has been dated to between 60 and 55 thousand years ago (ka) (17).

1.2.2 Tourtoirac rock shelter

The Tourtoirac rock shelter is located 40 km east-northeast of Périgueux on the middle terrace of the limestone cliffs situated on the left bank of the Auvezère river which runs through the Tourtoirac village. The site was briefly explored in the second half of the 19th century (18) and subjected to limited excavations (19) and looting until 2007. From 1968 to 1972, H. Laville excavated two trenches totaling ~24 m2 in the shelter at a maximum depth of 2 m without reaching the bedrock (20-22). In 2021, the archaeological investigations at the site and the study of the remains yielded since the first excavation were resumed by the multi-disciplinary team led by L. Doyon.

The reassessment of the stratigraphy exposed by Laville at the Tourtoirac rock shelter allows the description of three stratigraphic units (SU), from SU1 on top to SU3 at the bottom. The top half of SU1 preserves a record of Middle Gravettian occupations and is dated between 31 and 29 ka (23). Noaille and Raysse burins were found, although mixed in the same archaeological horizon, which suffered from glacial post-depositional deformations. The lower half of SU1 yielded numerous carnivore remains - fox, cave bear, wolf and hyena - with limited items of material culture. SU2 is almost sterile with few bird and mammal remains and some rare lithic flakes at its base. SU3 yielded a rich faunal assemblage alongside numerous stone tools. The faunal spectrum is dominated by reindeer (89.6% of NISP). Limb bones are overrepresented while axial elements are comparatively rarer. Cut marks are present on 31.9% of the faunal remains. Together, this pattern suggests the preferential introduction of meaty skeletal portions at the site. Over 30 bone retouchers were found in SU3, mainly on reindeer femur and tibia fragments. Technologically, the lithic assemblage suggests a highly fragmented operational sequence with the introduction of exhausted tools at the site, in situ production and repair of tools as well as preparation of tools that were carried away from the site. The lithic raw material mainly comes from deposits located in the Isle River valley and beyond within a 50 km distance, i.e., up to three days walking distance from the site. Diagnostic cultural features in the lithic assemblage suggest the attribution of the Middle Paleolithic artifacts to the Quina technocomplex with some Levallois elements. All radiocarbon samples from the lower half of SU1 to SU3 yielded results that exceed the limit of the method despite good collagen preservation.

A total of 22 human remains were found at the Tourtoirac rock shelter (24). Two were discovered by Laville in 1970 (21). Ten were identified during the screening of the faunal assemblage recovered by Laville and curated at the Musée national de Préhistoire. The remaining ten were recovered during the excavation that has resumed since 2021. A single *Homo sapiens* deciduous incisor was unearthed in association with Middle Gravettian lithic tools in the upper half of SU1. The other remains, i.e., one deciduous molar, two rib fragments, nine long bone (tibia, femur, fibula) fragments, two metacarpals, one proximal phalanx, two medial phalanges and four distal phalanges, are assigned to Neanderthal individuals based on diagnostic features or their robustness. Four remains were recovered from backfield sediments at the rear of the shelter. The six human remains found in contexts that were undisturbed by

looters or undeclared excavations were all found in SU3. Spatial correlation with those found during the Laville excavations supports their association with Middle Paleolithic cultural remains and suggests their deposition in the same stratigraphic unit. Although OSL and ESR dating are ongoing, the presence of Quina Mousterian and rare Levallois lithic artifacts at the site alongside a faunal assemblage dominated by reindeer suggests, based on our current understanding of the regional chrono-cultural sequence, a Neanderthal occupation likely spanning from the end of MIS 5 and throughout MIS 4. Here we successfully retrieved aDNA from a tibia (Fig. S6), a femur (Fig. S7) and a distal hand phalanx (DP5 left) (Fig. S8). Both long bones were identified in the faunal assemblage excavated by Laville now curated at the Musée national de Préhistoire, Les Eyzies, France. The distal phalanx was found in SU3 during the 2022 excavation. While stratigraphic correlations with Laville's excavations are still ongoing, all remains currently appear to come from a single phase of Neanderthal occupations at the site.

1.3 Serbia

1.3.1 Pešturina Cave

Pešturina Cave is situated near the Jelašnica Gorge, 20 km away from the city of Niš. It is the only Paleolithic archaeological site from the Central Balkans that documents both Neanderthal and modern human remains. In 2006, excavations revealed five stratigraphic layers spanning from the Bronze Age until the Middle Paleolithic (25). Three human skeletal elements were retrieved from three different layers. The first element, a partial atlas (Pes-1) was found at the bottom of Layer 2, which dates back to 31-29 ka, the second, a fragmented radial diaphysis (Pes-2), between Layer 3 and 4, dated at 38.9 and 92 ka. The fact that Pes-2 was located at the contact surface result into a long dating interval due to bioturbation effects (26). Finally, and most crucially for this study, a very well preserved permanent upper right molar labeled Pes-3 (Pešturina 3, Fig. S9) was discovered in Layer 4b along with artifacts attributed to the Quina Mousterian of Central European type (27, 25). Pes-3 presents typical Neanderthal morphological characteristics and has an estimated age of 102 ± 3,2 ka (27). A new ESR dating was performed on a tooth from the same stratigraphic layer as Pes-3 and yielded an estimate of around 111 ± 11 ka (25).

1.4 Germany

1.4.1 Sesselfelsgrotte

Sesselfelsgrotte is a rock shelter in the Altmühl Valley, Bavaria, excavated between 1964 and 1981 (28) and currently under re-investigation. The site preserved rich Middle and Upper Paleolithic occupations buried within approximately seven meters of stratified deposits. The Middle Paleolithic sediments yielded numerous animal bones, microfauna remains, fire residues – including potential hearths (28), and a total of 14 skeletal remains. These consist of two fragmented deciduous molars of 12-year-old children, from layers G2 and M2 (Sesselfeslgrotte 2 and 3), and the 10 fetal bones from G5 (Sesselfelsgrotte 1, 29). IR-OSL results from M1 to M3 yield an average age of ~73 ka (30), while previous as well as ongoing palaeoenvironmental studies indicate that these deposits formed during interstadial conditions. This phase was followed by the accumulation of a 1.5 m sequence (layers L to H), which saw a decrease in Neanderthal visits, and the first appearance of mammoth remains at the site, possibly reflecting an environmental deterioration (28). In contrast, the above-lying G1 to G5 document a phase of intensive Neanderthal site use associated with the Micoquian technocomplex (31) (> 8000 lithic artifact / m3 of excavated sediment), which took place during an interstadial roughly dating between 42 and 60 ka (30, 32).
Unlike the teeth, the fetal remains were identified only in the 1990s, long after their excavation in 1968-70 (29). Apart from the femur (Fig. S10) and the fibula, all the skeletal remains of Sesselfelsgrotte 1 were uncovered from layer G5 in square B7. The two long bone fragments were uncovered from a collapse of the west excavation profiles of squares B7 – Z7, where the G layers were exposed (28). Since their identification, these fetal remains have been interpreted as belonging to the same individual and the same layer (29), also given that detailed faunal analysis did not reveal other compatible skeletal elements from the neighboring units.
Given their limited vertical (max 15 cm) and horizontal (ca. 1 m) dispersion, the fetal remains have been interpreted as potentially the result of an intentional burial, highlighting complex social behaviors among Neanderthals at the site (29). This hypothesis, however, needs to be further supported by stratigraphic and taphonomic evidence.
The published thermal luminescence ages closer to the depositional context of the fetus come from layer G4 in square A7, and date to 51.1 ± 10.3 ka and 57.5 ± 12.8 ka (30). Based on the available geochronological data, we conclude that the Neanderthal fetus (Sesselfelsgrotte 1, located in layer G5), dates either to the very beginning of MIS 3 or to the very end of MIS 4.

2. Extraction and library preparation

DNA was extracted from bone and tooth powder following a modified version (33) of an established protocol (34), which was specifically designed to retrieve short DNA fragments. After DNA extraction, 30 µl of each extract were used to build single stranded and double indexed libraries following the manual and automated version of the Gansauge et al. (2020) protocol (35). Single stranded library preparation in ancient DNA is generally preferred over double stranded, because it allows the recovery of single stranded overhangs and fragments with a small average size. This protocol allows a highly efficient ligation strategy, which occurs by using randomly chosen adaptors, and a lower ligation bias when seven or eight degenerated bases are used. Furthermore, it increases library yields resulting in higher complexity of the samples as measured through qPCR (36). The use of the doubled indexing strategy allows for higher accuracy in the identification of sequences belonging to the corresponding sample in comparison to single indexed libraries, which can be problematic due to misidentifications caused by cross-contamination or issues during PCR amplification in bulk such as jumping PCRs (39). Most of the libraries were not Uracil-DNA-Glycosidase (UDG) treated to preserve the full damage profile and facilitate aDNA authentication as well as the filtering of reads with C to T substitutions for contaminated samples (37). Negative and positive controls were taken throughout the entire workflow. Libraries were then enriched for the entire mitochondrial DNA (mtDNA capture) with an in-solution capture approach based on synthesized probes (38) or generated from long-range PCR on present-day DNA (39) and later sequenced on an Illumina HiSeq4000 (1x75cyles) for up to ~70M reads.

3. MtDNA mapping and consensus reconstruction

The sequencing data from each run was merged and subsequently run through the EAGER pipeline (40). Reads were aligned to the Reconstructed Neanderthal Reference Sequence (RNRS) using CircularMapper (parameters –n 0.01, –l 16500, -q 30) as implemented in EAGER to account for the circular nature of mtDNA (40). We selected a mapping quality filter of 30 to remove non-uniquely mapping reads. PCR duplicates were discarded with DeDup to avoid overinflated coverages (40) and damage patterns were calculated with mapDamage2, a method to estimate the frequency of C to T substitutions at both ends of the mapped fragments (41) (Table S1 and Dataset S1). Using the program bamtofastq, the alignment (BAM) files were converted to FASTQ files. These were then mapped against the modern human reference sequence (rCRS) using CircularMapper (parameters –n 0.0001, –l 16500, -q 30) in EAGER. The resulting alignment (BAM) files were then used to estimate mtDNA contamination levels based on conditional damage pattern (contDeam) as well as against a reference database of 256 putative contaminant modern-day mtDNA sequences using schmutzi (42) (Table S2). In addition, this program reconstructs the mtDNA consensus sequence making use of deamination patterns, fragment length distributions and contamination estimates by probabilistically estimating the authenticity of each base at every position of the mtDNA sequence. A consensus FASTA file with bases having a schmutzi quality of at least 30 was then created for Goyet Q305-1 and Trou Magrite 2422-36. However, for individuals with levels of contamination above 10% we used a more conservative approach for the reconstruction of the consensus sequences. In order to restrict the analysis to putatively ancient DNA reads, we retained fragments that showed evidence of C-to-T mismatches at the first and/or last three positions (Customterminus 3) using the single stranded mode of PMDtools (37). We then downscaled the deaminated positions and trimmed the ones showing low base quality (<20) for up to three positions based on the directionality of each fragment. A consensus base at each position was called when covered by at least three DNA fragments and 65% of the fragments carried an identical base using Geneious Prime v.2022.1.1.

4. SNP analysis

This approach was employed due to the challenge in confidently identifying the phylogenetic placement of samples that have low mtDNA depth coverage because of the strong filtering due to high levels of contamination (Saint-Césaire RPB_117, Goyet D183-4, Tourtoirac N.1, Tourtoirac N.4, Tourtoirac_D_14 and Sesselfelsgrotte 1). After applying the same consensus procedures described in Section 3, these samples resulted in between 1,000 and 5,000 unidentified positions. This high level of missing data prevents a robust phylogenetic position using a Maximum Parsimony approach. To systematically evaluate derived/ancestral allele states and potential contamination across ancient individuals, we developed a new custom Python pipeline that:

1. Identifies node-specific SNPs:
    a. Parses a multiple sequence alignment of published Neanderthal genomes, using the rCRS as the reference.
    b. Using a Maximum Parsimony tree as a visual reference (Fig. S11), for each predefined node (Table S3), it extracts positions where all samples deriving from that node share the same allele and differ from the defined outgroups, after excluding ambiguous positions or alignment gaps.
    c. Progressively moves along the phylogeny, first using the modern human reference sequence (rCRS) as an outgroup to identify positions with derived alleles shared by all Neanderthals included in the alignment. Then, uses rCRS and Hohlenstein-Stadel as outgroups to identify positions with derived alleles shared by all Neanderthals included in the alignment other than Hohlenstein-Stadel, and continues until considering positions with derived alleles shared only among sub-lineages of the main Neanderthal mtDNA branch.
2. Analyzes sequencing data:
    a. Identifies the positions (Dataset S2C) within the alignment (BAM) file of each sample, for both pre- (Fig. S12-17A) and post-mortem damage filtered (Fig. S12-17B) data.
    b. Quantifies strand-specific metrics (number of forward/reverse mapping reads) at each SNP.
    c. Implements a conservative handling of putative damage as in Meyer et al. 2016 (44):
        i. Excludes forward-mapping reads if either the ancestral or derived allele is C (to avoid C→T damage).
        ii. Excludes reverse-mapping reads if either the ancestral or derived allele is G (to avoid G→A damage).
    d. Calculates the number and proportion of reads supporting the derived allele to identify correct placement along the phylogeny.

5. Pairwise nucleotide differences

To investigate the mtDNA diversity between different Neanderthal groups, we analyzed pairwise nucleotide distances among individuals divided in four groups: main Late Neanderthal mtDNA branch (n=17), Vindija (n=4), Goyet (n=5), and Feldhofer (n=2). Two multiple genome alignments of Late Neanderthals (including three newly generated sequences) were created: one with the complete mtDNA and the other with the coding region only. Pairwise distances were computed on the number of nucleotide differences and missing or ambiguous data, and gaps were removed to avoid discrepancy in the calculations. The pairwise distance matrices for each group were then transformed into long format, after removing self-comparisons and missing values. The resulting data consisted of pairwise distance values for each individual, annotated with their respective group. To test whether the number of nucleotide differences among sequences in the main Late Neanderthal mtDNA branch differs significantly from those in site-specific Late Neanderthal groups, we performed pairwise comparisons using the Wilcoxon rank-sum test (also known as the Mann–Whitney U test). This non-parametric test was chosen over parametric alternatives (e.g., t-test or ANOVA) due to the non-normal distribution of the data, which was evident in exploratory plots and supported by Shapiro-Wilk normality tests ($W = 0.9146$, $p = 5.14 \times 10^{-8}$). Moreover, ANOVA assumes homogeneity of variance and normality of residuals, which were not met in our dataset. The Wilcoxon test does not require these assumptions and is thus more appropriate for this type of genetic distance data. Since multiple pairwise comparisons were made (whole Late Neanderthal branch vs each of the other three groups), we applied the Benjamini–Hochberg (BH) procedure to adjust the p-values for multiple testing and to control the false discovery rate. None of the comparisons reached statistical significance after correction, however, the comparison with Vindija yielded a marginally significant trend, indicating possible subtle differences in pairwise nucleotide diversity between individuals analyzed from Vindija and the Later Neanderthals group (Table S4).

Goyet is the archaeological site with the highest number of Neanderthal mtDNA sequences studied up to date. We examined pairwise distances among sequences based on the number of nucleotide differences to test for similarities within and between the previously published and newly generated mtDNAs. In this analysis, we included all nine Goyet sequences (except Goyet D183-4 due to the high number of unidentified sites) and we deleted all ambiguous positions using MEGA X (45). Given that the D-loop is known to accumulate more substitutions due to its higher

mutation rate, we computed the pairwise distances for both the entire mtDNA and the coding region only. The results are consistent between the whole mtDNA and the coding region of the nine Goyet sequences (Table S5). Furthermore, the pairwise distance is in accordance with the phylogenetic sub-clustering among Late Neanderthals. More specifically, Goyet Q305-1 and Goyet Q305-4 belong to the same branch and have the lowest number of pairwise differences between them. In addition, the mtDNA of Goyet 2878-2D is indistinguishable from Goyet Q56-1, Goyet Q374a-1 and Goyet Q305-7, the latter three belonging to the same individual, as is the case for specimens Goyet Q57-1, Goyet Q57-2 and Goyet Q57-3 that represent another individual (46).

6. Phylotemporal distribution

To investigate the geographic, temporal, and phylogenetic structure of Neanderthal mtDNA variation, we compiled 49 previously published Neanderthal mtDNA sequences with ≥85% genome completeness, together with four newly generated high-coverage sequences. Phylogenetic relationships were inferred using the Maximum Parsimony method, applying partial deletion at a 90% threshold and performing 500 bootstrap iterations to evaluate node support. The resulting topology was color-coded according to the major mtDNA clades identified in the tree (Fig. S21A).

Archaeological site locations were georeferenced based on published coordinates. Each site is represented by circles scaled to the number of individuals with available mtDNA data. The dataset was divided into two temporal groups: individuals dated to Marine Isotope Stages (MIS) 5–4 and those from MIS 3 (Figs. S21B-C). Due to the broad chronological ranges of several archaeological contexts, it was not always possible to confidently assign sites to either MIS 5 or 4; these have therefore been combined into a single MIS 5–4 category. Circle colors correspond to the mtDNA lineage assigned to each individual, following the phylogenetic classification established in Fig. S21A.

The spatial and temporal distribution of mtDNA lineages (Fig. S21) reveals a marked reduction in genetic diversity between MIS 5–4 and MIS 3. Multiple distinct mtDNA lineages were present across Eurasia during MIS 5–4, whereas by MIS 3, a major Late Neanderthal lineage appeared, with two additional lineages geographically restricted to southwestern France. This reduction in mtDNA diversity suggests a substantial demographic turnover in the genetic history of Late Neanderthal populations.

7. BEAST analyses

In the Bayesian statistical framework implemented in BEAST2 we selected Bayesian Skyline Coalescent as tree prior, a Markov Chain Monte Carlo (MCMC) method used for demographic inferences deriving from sequence data. The method assumes a panmictic population, meaning that all members belonging to this population have the same probability of contributing genetically to the next generation. Considering the geographical and temporal range of Neanderthals, and the low sample size from single locations, the presence of population structure in our dataset is almost certain. However, with the steady increase in the number and quality of Neanderthal mtDNA sequences, it is becoming possible to provide more accurate estimations (47). Since BEAST relies on Bayesian inference, the choice of priors can significantly influence posterior estimates, particularly when sequence data is limited, or model complexity is high. To assess whether the priors used in our analysis were overly restrictive (risking overfitting) or too diffuse (providing insufficient constraint), we conducted a "sample from prior" analysis. These performed runs excluded sequence data and sampled solely from the prior distributions specified for parameters such as specimen ages and tree topology. Because the tree topology was fixed throughout all analyses, these tests allowed us to isolate the impact of priors while controlling for phylogenetic structure.

7.1 Quantitative comparison of prior and posterior distributions

To assess the informativeness of the sequence data, we compared the prior and posterior distributions under both strict and relaxed log normal clocks as well as with and without Thorin tip-date. In the strict clock analysis and excluding Thorin as a tip date, the posterior mean log-likelihood was -30,947.21 with a narrow 95% HPD interval [-31,002.58 to -30,894.56] and a density peak around 0.035 with low standard deviation. In contrast, the prior had a substantially different distribution with a posterior mean of -1,727.35 [95% HPD: -1,745.05 to -1,705.57] and a slightly higher density peak (0.04) but with greater standard deviation. The sample-from-prior version of the same model produced a posterior identical to the prior: a mean of -1,595.23 [95% HPD: -1,719.21 to -1,443.79] with a density peak of 0.01 and a noticeably higher standard deviation. The posterior and prior likelihoods for the same analysis using the log-normal relaxed clock are highly similar (Table S6). These differences confirm that the data are clearly informative,

shifting the posterior away from the prior in both central tendency and uncertainty, indicating no overfitting from overly restrictive priors.

Similar results were found when Thorin was included as a tip date. The strict model posterior was nearly identical to the previous run [-30,947.98; 95% HPD: -31,004.64 to -30,890.09], with the prior again much lower [1,727.74; 95% HPD: -1,777.59 to -1,668.26]. In the corresponding sample-from-prior run, the posterior matched the prior [-1,592.83; 95% HPD: -1,727.12 to -1,412.69], both showing broader uncertainty and lower density (0.01), reinforcing the evidence that priors were not overly informative or constraining (Table S6).

7.2 Impact of tip dating on molecular age estimates

We evaluated how including or excluding tip dates affects molecular age estimates, focusing on two specimens: Les Cottés Z4-1514 and Thorin. These individuals are considered as Late Neanderthals based on direct dates and/or archaeological contexts, yet they occupy more basal positions in the phylogeny compared to the main Late Neanderthal mtDNA branch. When Thorin is left undated, its estimated age aligns with that of MIS 5-4 Neanderthals and is not overlapping with its indirect date estimate (~50 ka). This suggests that either (1) the published radiocarbon-based chronology of Thorin's is incorrect or, (2) the lineage leading to Thorin has a considerably slower mutation rate that results in an artificially older molecular date.

When a uniform tip date based on Thorin's indirect radiocarbon estimate is used as time anchor, it results in a localized influence on the molecular date of specimens clustering closely to Thorin such as Pešturina 3, Stajnia S500, Mezmaiskaya 1 and Mezmaiskaya 3. While their molecular dates became younger, they did not reach Thorin's radiocarbon date.

In contrast, Les Cottés Z4-1514, which has a direct radiocarbon date securely falling within the Late Neanderthal time interval, maintained broadly consistent age estimates regardless of whether it was considered as dated or undated. When treated as undated, its molecular age estimate resulted in a slightly older mean value but with a 95% HPD interval overlapping with its radiocarbon date. This consistency suggests that it is feasible to obtain reliable molecular dates also for Neanderthal sequences falling more basally than the main Late Neanderthal mtDNA branch. However, we observe a tendency to overestimate the tip age of more basal lineages and future calibration points on these sections of the phylogeny will be pivotal to producing more accurate molecular date estimates.

7.3 Effect of clock model on undated specimens

We further compared strict and relaxed clock models and found notable differences in estimated ages and uncertainty, particularly for undated specimens. The relaxed clock log-normal model yielded generally older molecular age estimates with wider HPD intervals. This is expected given the model's allowance for lineage-specific rate variation, which broadens posterior uncertainty.

7.4 Tip-to-Root analysis

To independently assess the presence of a temporal signal in our data and validate the molecular dating results obtained with BEAST2, we implemented a tip-to-root regression analysis using TempEst (48). A Maximum Parsimony tree was computed using the alignment of Neanderthal coding-region mtDNA sequences, and the tree was rooted either using the modern human reference (rCRS) or the Hohlenstein-Stadel (HST) sequence. To quantify temporal structure, we computed the genetic distance between each Neanderthal sequence and the root (rCRS or HST) and transformed this into an estimated age using the mutation rate specific to the coding region of mtDNA (1.57×10^{-8} substitutions/site/year, 38). Given the coding region length of 15,445 bp, this results in one mutation every 4124 years on average. We first identified the youngest individual in the phylogeny as a baseline and subtracted its distance from that of all other individuals, obtaining relative distances to root. These differences were then multiplied by 4124, converting substitution distances into years before present (Dataset S2B).

7.5 Integrative dating approaches

Overall, our findings suggest that the priors used in our BEAST analyses were broad enough to allow meaningful inference without overfitting, as demonstrated by the divergence between prior and posterior distributions, especially in runs with informative sequence data. At the same time, molecular data alone cannot fully resolve dates, especially for undated individuals falling more basally than existing dated specimens. This reinforces the importance of integrative approaches combining molecular, archaeological and chronological data to reconstruct robust chronologies. The influence of both tip dates and the choice of the clock model should be considered carefully in

interpreting molecular age estimates, particularly when calibrating chronologies in paleogenomic datasets with uneven temporal coverage.

8. Distribution map generation from ROAD data

To compile a comprehensive dataset of Neanderthal-associated archaeological sites, we employed the recently developed and publicly available querying interface "AskROAD" (https://www.roceeh.uni-tuebingen.de/askROAD/), which facilitates user-driven access to ROAD (ROCEEH Out of Africa Database). This tool allows for the construction of complex, multi-step database queries through an intuitive graphical interface that guides users through spatial, chronological, and cultural parameters. The ability to tailor searches dynamically makes AskROAD particularly well-suited for paleoanthropological and archaeological research involving large-scale spatial and temporal datasets. We constructed three distinct but complementary queries, each designed to capture different facets of Neanderthal archaeological presence. Below are the queries outlined in more detail so that, for reproducibility, it is possible to query the ROAD database in the same fashion:

Query 1

- Step 1a
    - Continent -> Europe
- Step 2
    - Age MIN -> 60.000
    - Age MAX -> 130.000
- Step 3b
    - Culture -> Middle Paleolithic

Query 2

- Step 1a
    - Continent -> Europe, Asia
- Step 2
    - Assemblage Category -> Human remains
    - Age MIN -> 40.000
    - Age MAX -> 130.000
- Step 3a
    - Species -> neanderthalensis

Query 3

- Step 1a

- Subcontinents -> Central Asia, Western Europe, Southern Europe, Northern Europe, Island Europe, Eastern Europe, Central Europe, Caucasus.

· Step 2

- Age MIN -> 40.000
- Age MAX -> 130.000

· Step 3b

- Technocomplex -> Mousterian Eurasia, Mousterian of Acheulean Tradition, Denticulate Mousterian, Late Mousterian, Micoquian, Châtelperronian.

The first query was aimed at identifying sites within Europe that are dated between 130,000 and 60,000 years ago. This time frame corresponds to a period prior to the widespread dispersal of *Homo sapiens* into Europe and is therefore critical for isolating contexts primarily associated with Neanderthal populations. The second query expanded the geographic scope to include Europe, Asia, and Africa, and focused on the presence of human remains dated between 130,000 and 40,000 years ago, with a specific filter applied to fossils identified as *Homo neanderthalensis*. This allowed us to capture all known sites within the ROAD database where Neanderthal skeletal remains have been documented. The third query further refined the cultural dimension by targeting technocomplexes typically associated with Neanderthals—namely Mousterian, Micoquian, and Châtelperronian traditions (despite the latter association being under discussion in the literature)—across a broad swath of regions including Central Asia, the Caucasus, and various subregions of Europe (e.g., Western, Eastern, Southern, and Island Europe), within the date range of 130,000 to 40,000 years ago. Given the broad spatial and temporal scope of this study, "Mousterian" was adopted as the highest-level techno-cultural classification, as it offers a consistent and comprehensive framework for characterizing the archaeological record across both dimensions.

Together, these three queries were designed to create a robust and multi-dimensional dataset that reflects not only the biological presence of Neanderthals through fossil evidence but also their cultural and technological signatures. Each resulting query produced a dataset that could be downloaded in tabular format (.csv), which was subsequently imported into the R statistical environment for further processing. Within R, the datasets were merged and harmonized, and we conducted a thorough data-cleaning procedure to ensure analytical reliability. This included filtering records based on chronological precision, retaining only entries with estimated date ranges between 2,000 and 30,000 years. Such filtering is essential for meaningful temporal analyses, as excessively broad age estimates can produce false signals of consistent Neanderthal presence in a region and obscure changes over time.

To enable diachronic investigation of spatial patterns, the cleaned dataset was divided into discrete time slices of 10,000 years. This temporal resolution strikes a balance between capturing long-term trends and maintaining sufficient site counts for statistical robustness. These time slices served as the basis for subsequent spatial analyses. To visualize the spatial distribution of Neanderthal-associated archaeological sites, we employed Kernel Density Estimation (KDE). This statistical method creates a continuous surface representing the density of points across geographic space by applying a smoothing function—typically a Gaussian kernel—centered on each site location. The influence of each site decreases with distance, allowing areas with tightly clustered data points to emerge as regions of higher density. The resulting heatmaps provide an interpretable representation of spatial intensity, with "hotspots" indicating zones where Neanderthal presence was most concentrated. This approach enables the identification of regional patterns and potential activity centers across different time slices, facilitating a deeper understanding of Neanderthal spatial behavior and occupation dynamics over time. This integrated approach allowed us to construct a nuanced picture of Neanderthal occupation dynamics across both time and space, grounded in systematically retrieved and reproducibly filtered data from one of the most comprehensive archaeological databases currently available.

9. Getis–Ord Gi* statistics

9.1 Methods — Spatial statistics and rationale

The analyses of the ROAD dataset used kernel density estimates (KDEs) to illustrate temporal changes in Neanderthal site density. While KDEs are useful for visualizing where sites are concentrated, they are purely descriptive and sensitive to spatial autocorrelation and uneven research intensity. Because archaeological discovery, preservation, and excavation effort vary markedly across Europe, apparent density peaks may reflect sampling bias rather than true behavioral patterning. To address these limitations, we applied a formal spatial–statistical approach using the local Getis–Ord Gi* statistic, which tests whether each location lies within a neighborhood of unusually high or low values compared to the overall mean. Positive Gi* z-scores indicate statistically significant clusters of high site density ("hotspots"), whereas negative scores represent low-density clusters ("coldspots"). This method provides a reproducible test of local clustering that explicitly accounts for spatial dependence and corrects for multiple comparisons.

We divided the study area into a regular hexagonal grid with cells approximately 50 km across and examined how clustering changed through a series of 10-thousand-year time slices spanning 130–40 ka. Sites whose age ranges overlapped with a given slice were counted into their respective hex cells. Each cell's neighborhood was defined as all cells within 150 km, allowing local comparisons between nearby areas. For every time slice, we identified statistically significant clusters of high site density through randomization tests based on 999 permutations, applying a false discovery rate (FDR) of 0.10 to control for multiple testing. We also calculated standardized Gi* z-scores to measure the intensity of clustering and to quantify change through time (ΔGi*).

Three complementary analyses were performed:

1. Slice-wise hotspot summaries that describe the total number and areal extent of hotspots in each time slice, providing a temporal overview of clustering.

2. Change through time was examined in two ways: (a) baseline-anchored contrasts, where each slice was compared to the 130–120 ka baseline to classify hexes as persisting, newly emerging, or lost hotspots; and (b) rolling slice-to-slice contrasts, comparing each time interval with the immediately preceding one to trace spatial dynamics across time. Overlap between periods was quantified with Jaccard similarity and average Δgi*.

3. Structured permutation tests assessed whether the apparent more recent concentration of Neanderthal sites in France could be explained by sampling bias. Keeping the spatial distribution of sites fixed, we randomly reassigned "Baseline" (130–120 ka) and "Current" slice labels among points and recalculated the number of new hotspots in France ($z \geq 1.96$) in each of 2,000 permutations. The resulting p-values measure how often an equal or greater number of new hotspots in France would occur by chance, given the existing spatial pattern. As a complementary diagnostic, additional slice-wise permutation tests randomly reassigned per-slice site counts to hex cells, evaluating whether observed hotspot counts exceeded random expectations.

Together, these analyses provide: (i) a statistically grounded identification of clustering with FDR control; (ii) transparent visualizations of hotspot emergence, persistence, and loss; and (iii) explicit hypothesis tests that account for spatial sampling, addressing previous concerns that KDE-based interpretations lacked statistical inference and were sensitive to spatial–temporal autocorrelation.

9.2 Results — Temporal and spatial trends

The slice-wise hotspot summaries (Table S10) show a contraction in overall hotspot area from 130–100 ka, followed by renewed expansion after ~90 ka. Hotspots occupied 152 cells at 130–120 ka, increased slightly at 120–110 ka (179), and declined sharply at 100–90 ka (66). Thereafter, both hotspot counts, and area grew steadily, peaking during 80–70 ka and 70–60 ka, and remaining high into the 60–50 ka and 50–40 ka slices.

Rolling, slice-to-slice contrasts (Fig. S22) reveal how this pattern developed. Between 130–120 ka and 120–110 ka, most baseline hotspots persisted while a moderate number of new clusters emerged. The 110–100 ka interval saw widespread losses, suggesting a temporary fragmentation of the spatial network. From 100 ka onward, new hotspots increasingly outnumbered lost ones, and Jaccard overlap stabilized, indicating spatial consolidation rather than random turnover.

Baseline-anchored contrasts (Fig. S23) emphasize longer-term change relative to the earliest phase. Early slices (120–110 ka) closely resemble the baseline (Jaccard ≈ 0.85), but 110–100 ka and 100–90 ka display extensive loss of baseline hotspots and limited persistence. From 90 ka onward, the balance reverses: most new hotspots appear in western and central Europe, and by 80–60 ka the hotspot footprint is both larger and geographically more focused than in the initial phase.

The structured permutation tests (Table S11) confirm that this late concentration in France exceeds expectations under random spatial sampling. Early intervals (120–100 ka) yield $p \approx 1.0$, consistent with no new hotspots in France. From 100–90 ka onward, observed values surpass the random expectation, and p-values decline steadily. The 60–50 ka ($p = 0.012$) and 50–40 ka ($p = 0.038$) slices are statistically significant, indicating that the increase in French hotspots is unlikely to result from sampling bias alone.

Finally, the slice-wise permutation tests (Table S12) show that early slices fall within the expected range of the null model, while later slices trend above it. These results reinforce both the descriptive increase in hotspot counts and the inferential evidence for a genuine geographic concentration of Neanderthal activity towards the end of their existence.

9.3 Summary on statistical inference and robustness

Although spatial density maps can visually highlight areas with many archaeological finds, such patterns alone do not demonstrate whether the clustering is statistically meaningful. Apparent concentration may arise from uneven excavation, preservation, or research effort. The Getis–Ord Gi* framework used here provides a formal test for whether observed clusters of Neanderthal sites are significantly denser than would be expected from random spatial sampling. The analysis identifies local “hotspots” where each cell is surrounded by neighbors with consistently high site counts, while controlling for multiple comparisons and the spatial structure of the data. Because the spatial scale (50 km hexes) and neighbor distance (150 km) are held constant, the method highlights relative clustering rather than absolute counts, identifying areas that stand out statistically from their surroundings.

The slice-wise permutation tests assess each time slice independently, comparing the observed number of hotspots with the distribution expected if site counts were randomly reassigned among cells. Early and middle slices (130–90 ka) fall within the 5–95 % range of their null distributions, whereas slices younger than about 80 ka show systematically higher values, consistent with a genuine intensification of clustering. The structured permutation tests further address spatial bias by randomizing the temporal labels of fixed site locations. This evaluates whether the observed rise in new hotspots in France could emerge by chance from the existing spatial sample. Significant results for the 60–50 ka and 50–40 ka intervals ($p = 0.012$ and 0.038) indicate that the late concentration of Neanderthal activity in France is unlikely to result from sampling bias alone. Together, these complementary tests show that Neanderthal-associated assemblages became increasingly spatially focused on western Europe after ~80 ka, reflecting genuine demographic persistence rather than uneven discovery.

10. Subsampling test of geographic spread

To test whether the apparent late-stage geographic expansion could be driven solely by increased sample size, we conducted a locality-level rarefaction analysis within a fixed spatial window (10°W-45°E; 30°-60°N). We used the 80-70 ka time slice as a baseline and downsampled the younger slices (70-60 ka, 60-50 ka, and 50-40 ka) to match the number of localities observed in the 80-70 ka slice ($n = 60$), repeating the subsampling 1000 times. We quantified longitudinal spread using two complementary metrics: (i) the longitudinal range covered (min-max longitude and a robust central 95% range), and (ii) the number of occupied longitudinal bins (2° wide) and the appearance of bins not present in the 80-70 ka baseline.

Under rarefaction, the 70-60 ka slice exhibited a similar full longitudinal range to the 80-70 ka baseline (median = 47.2° in both cases; one-sided Monte Carlo p = 0.502), but showed a significantly broader central 95% longitude range (median = 40.0° vs 32.3°; p = 0.009) (Table S13), indicating an early widening of the core geographic distribution. The 60–50 ka slice showed a significantly greater full longitudinal range than the 80-70 ka baseline (median = 51.9° vs 47.2°; p = 0.045), accompanied by a significantly larger central 95% range (median = 43.5°; p = 0.011). The 50-40 ka slice displayed the strongest signal of expansion, with significantly larger values for both the full longitudinal range (median = 51.9°; p = 0.022) and the central 95% range (median = 47.0°; p = 0.004) relative to the 80-70 ka baseline. In parallel, all post-80-70 ka slices consistently occupied new longitudinal bins absent from the baseline distribution under rarefaction (median = 2 new bins for 70-60 ka and 3 new bins for both 60-50 ka and 50-40 ka), indicating a progressive broadening of the west-east spatial footprint even when sample size is held constant. Together, these results support a post-80-70 ka widening of geographic distribution that cannot be explained solely by increased sampling intensity.

11. Reproducing the analysis of Yaworsky et al. 2024

As part of our study, we also reproduced the analysis conducted by Yaworsky and colleagues (2024) (57). The code used for this original study is openly available on Zenodo (https://zenodo.org/records/10809139) making it fully reproducible. Furthermore, the data that was used comes from ROAD, and was queried using dynamic PHP scripts. When these scripts are run anew, any new additions to the ROCEEH ROAD data will be included as well. This made it possible to re-run their analyses using the current, extended ROAD dataset. Our re-analysis incorporates a larger sample, increasing the number of archaeological sites from n = 94 in the original study to n = 131 (Fig. S24).

The purpose of the original study by Yaworsky and colleagues (2024) was to explore the climatic niche space of Neanderthals, both the fundamental niche and geographically projected potential niche space. By re-running their analyses (Fig. S25), we can confirm their findings as well as relate them to our own results. Here, we find a general decline in the projected potential niche space of Neanderthals following the last interglacial period, Marine Isotope Stage (MIS) 5e. This contraction becomes most pronounced around 65,000 years ago, during the peak of MIS 4—a glacial period marked by colder and drier climatic conditions. The timing and nature of this ecological decline align closely with our archaeological analysis of Neanderthal site distributions. Between 130,000 and 80,000 years ago, the spatial pattern of cultural presence in Europe was characterized by fluctuations and multiple regional concentrations. However, from approximately 80,000 years onward, we observe a notable contraction in the geographic distribution of Middle Paleolithic archaeological assemblages. This reduction culminates in a distinct density hotspot in southern France between 70,000 and 60,000 years ago, indicating a potential demographic and/or cultural refugium during a period of environmental stress.

Following this period, while the southern France hotspot remains relatively stable in terms of site density, the broader spatial pattern shifts again. A greater number of individual sites appear dispersed across much of western Eurasia, suggesting a phase of geographic re-expansion or diversification in occupied territories.

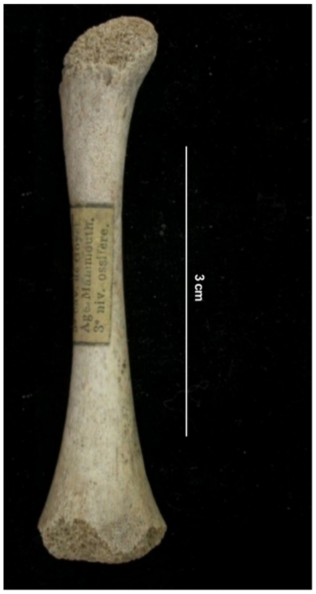

Fig. S1. Goyet Q305-1, right neonate femur, anterior view, Goyet (Troisième caverne), Belgium.

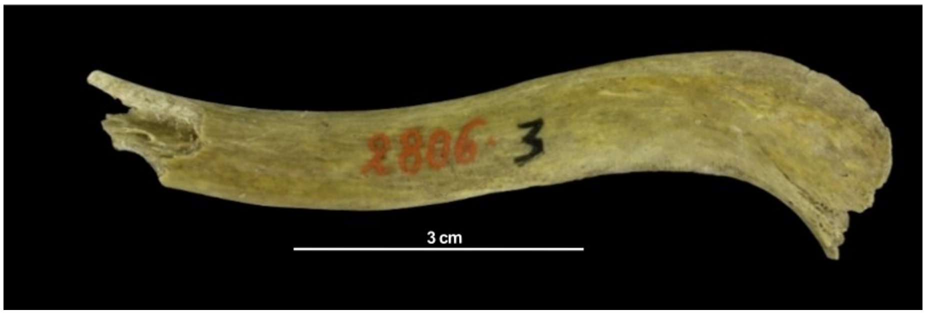

Fig. S2. Goyet D183-4, partial left clavicle, superior view, Goyet (Troisième caverne), Belgium.

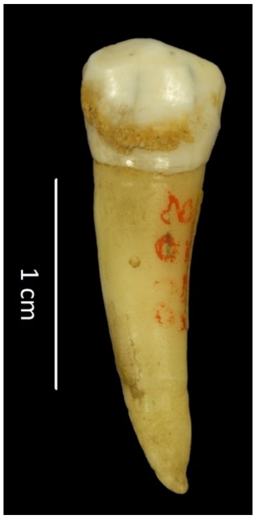

Fig. S3. Goyet 2878-2D, lower left second premolar, buccal view, Goyet (Troisième caverne), Belgium.

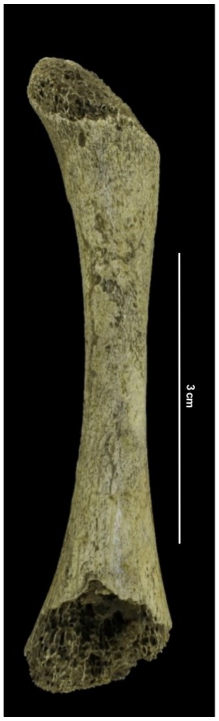

Fig. S4. Trou Magrite 2422-36, left neonate femur, anterior view, Trou Magrite, Belgium.

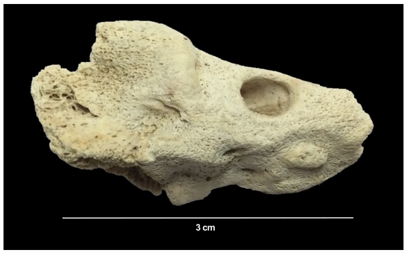

Fig. S5. Saint-Césaire RPB_117, left petrous bone, medial view, La Roche-à-Pierrot, Saint-Césaire, France.

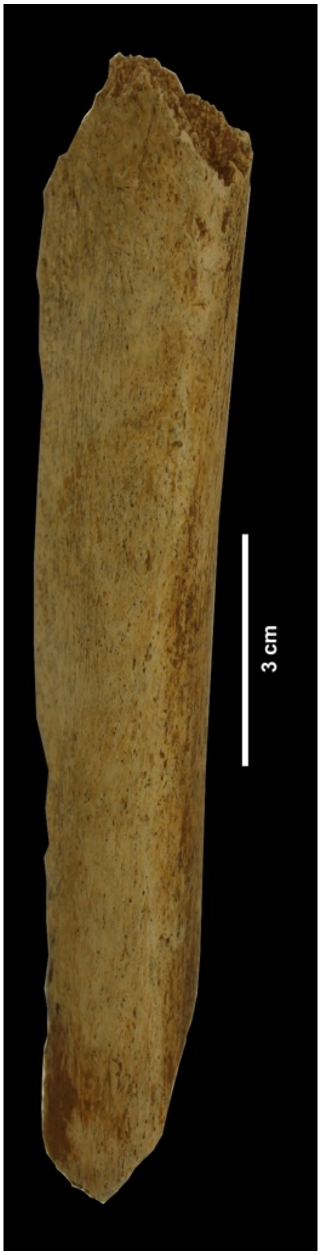

Fig. S6. Tourtoirac N.1 (F7 layer 5), left tibia, anterior view, Tourtoirac rock shelter, France.

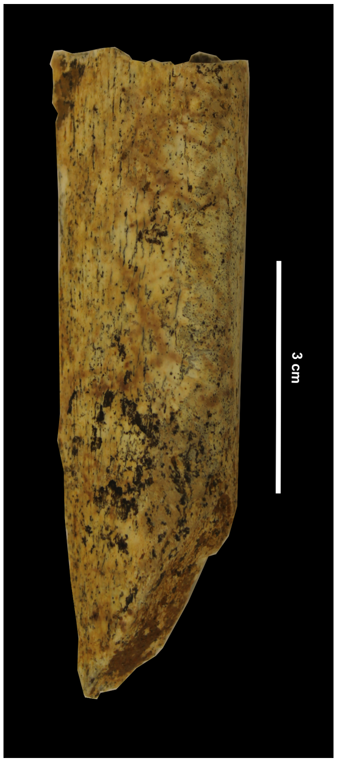

Fig. S7. Tourtoirac N. 4 (F7 layer 7), femur, anterior view, Tourtoirac rock shelter, France.

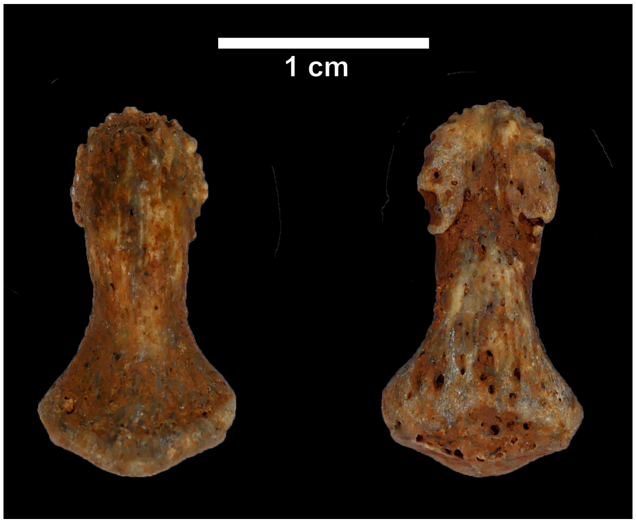

Fig. S8. Tourtoirac_D_14 (D5 A, US 3), distal hand phalanx (DP5 left), dorsal (left) and palmar (right) views, Tourtoirac rock shelter, France.

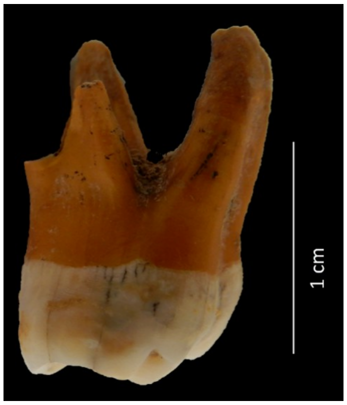

Fig. S9. Pešturina 3, upper right first permanent molar, mesial view, Pešturina Cave, Serbia.

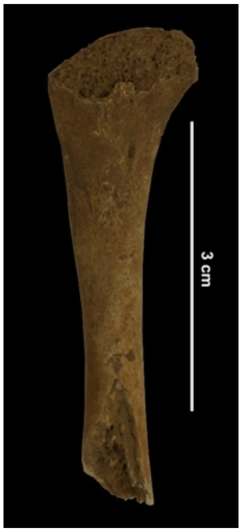

Fig. S10. Sesselfelsgrotte 1, right femur, anterior view, Sesselfelsgrotte, Germany.

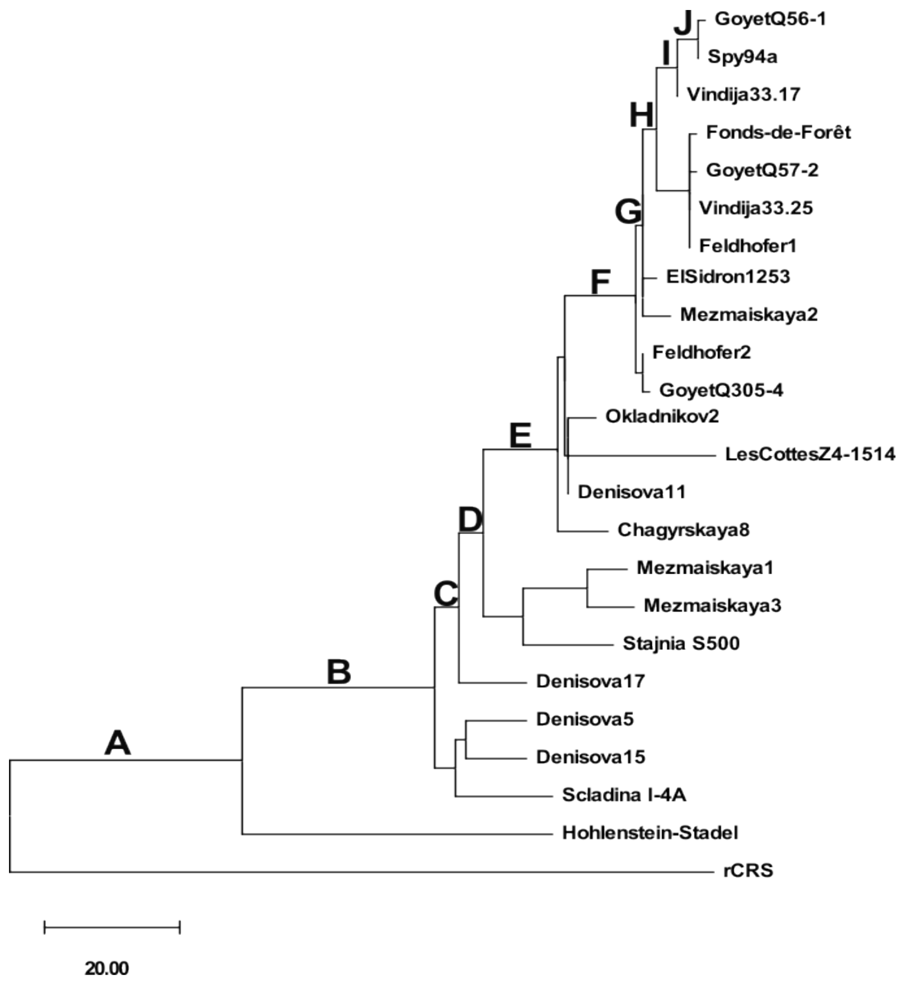

Fig. S11. Visual representation of nodes for SNP analysis.

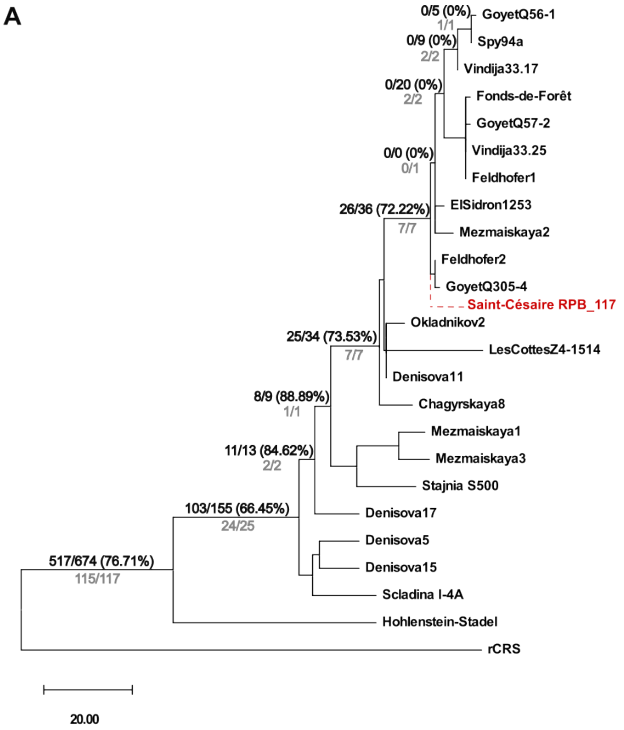

Fig. S12. Saint-Césaire RPB_117 phylogenetic positioning according to the SNP analysis before (A) and after (B) PMD filtering. Values in black represent the number of reads supporting the derived allele, relative to the total number of reads covering the defining SNPs for each node, and the resulting percentage in graphs. Values in gray indicate the number of SNP positions covered by at least one read within the node, relative to the total number of SNPs defining that node.

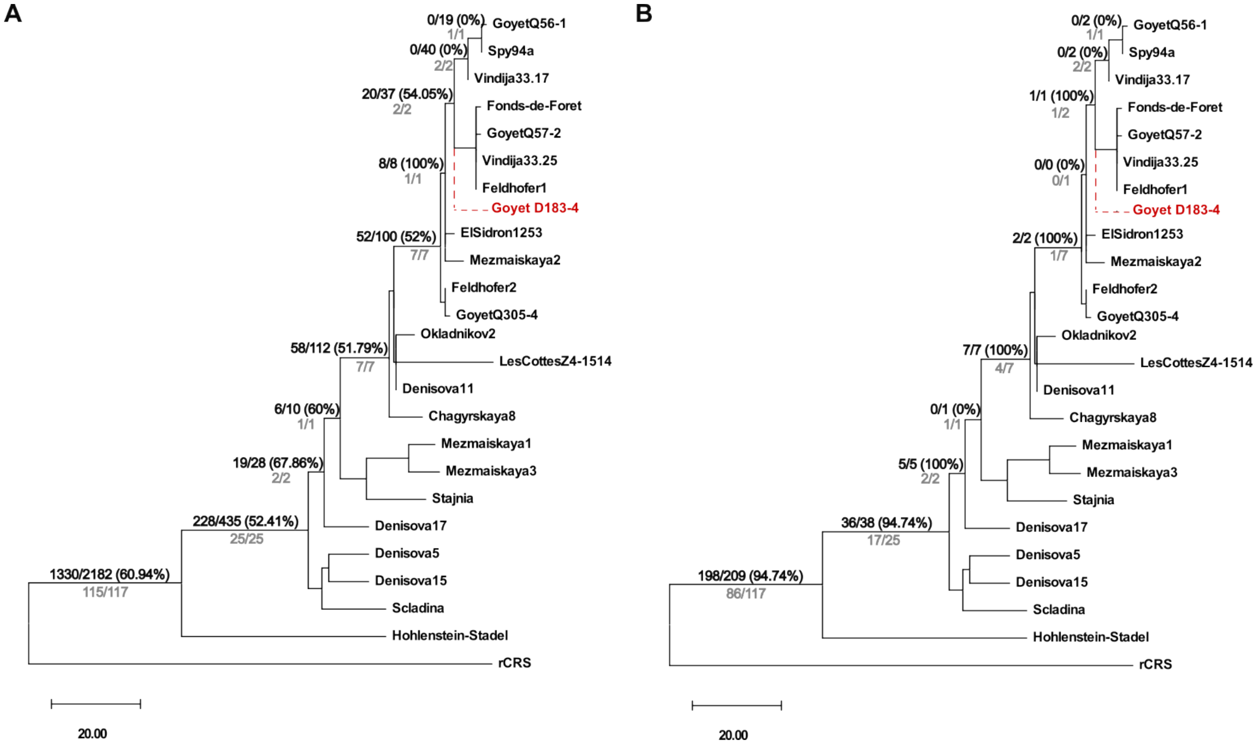

Fig. S13. Goyet D183-4 phylogenetic positioning according to the SNP analysis before (A) and after (B) PMD filtering. Values indicated above and below the nodes are as in Fig. S12.

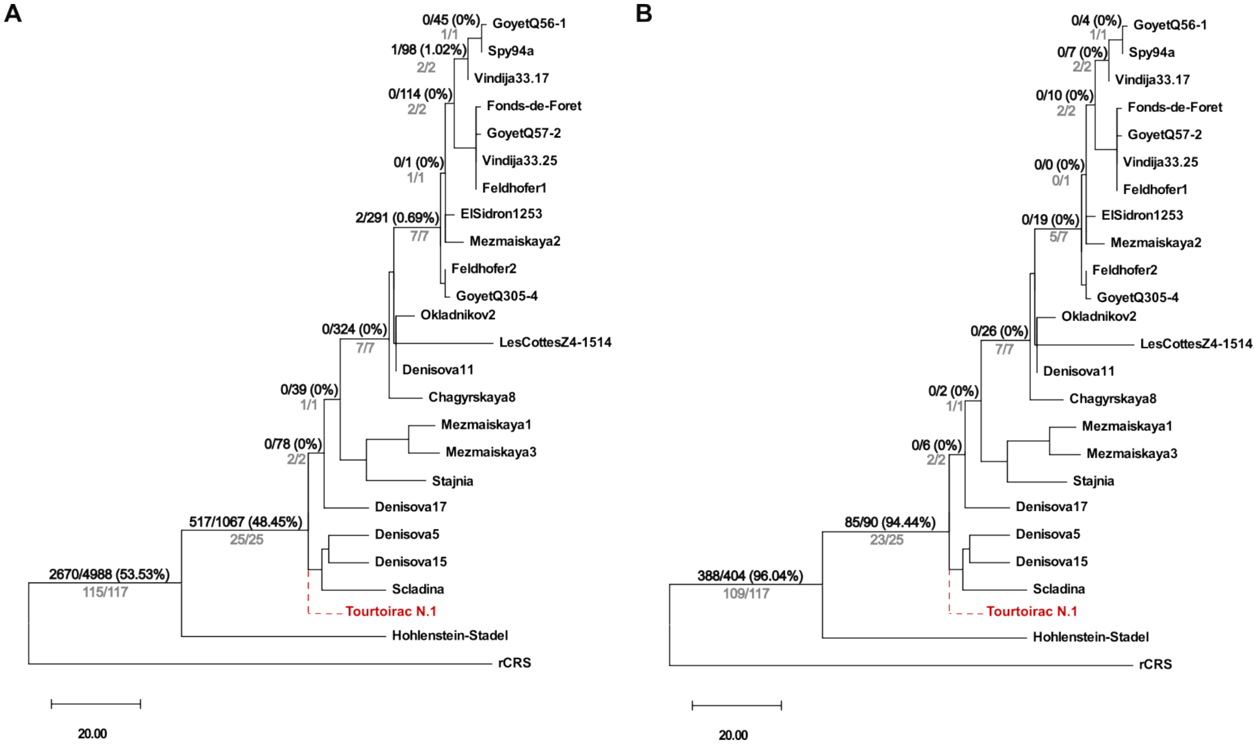

Fig. S14. Tourtoirac N.1 phylogenetic positioning according to the SNP analysis before (A) and after (B) PMD filtering. Values indicated above and below the nodes are as in Fig. S12.

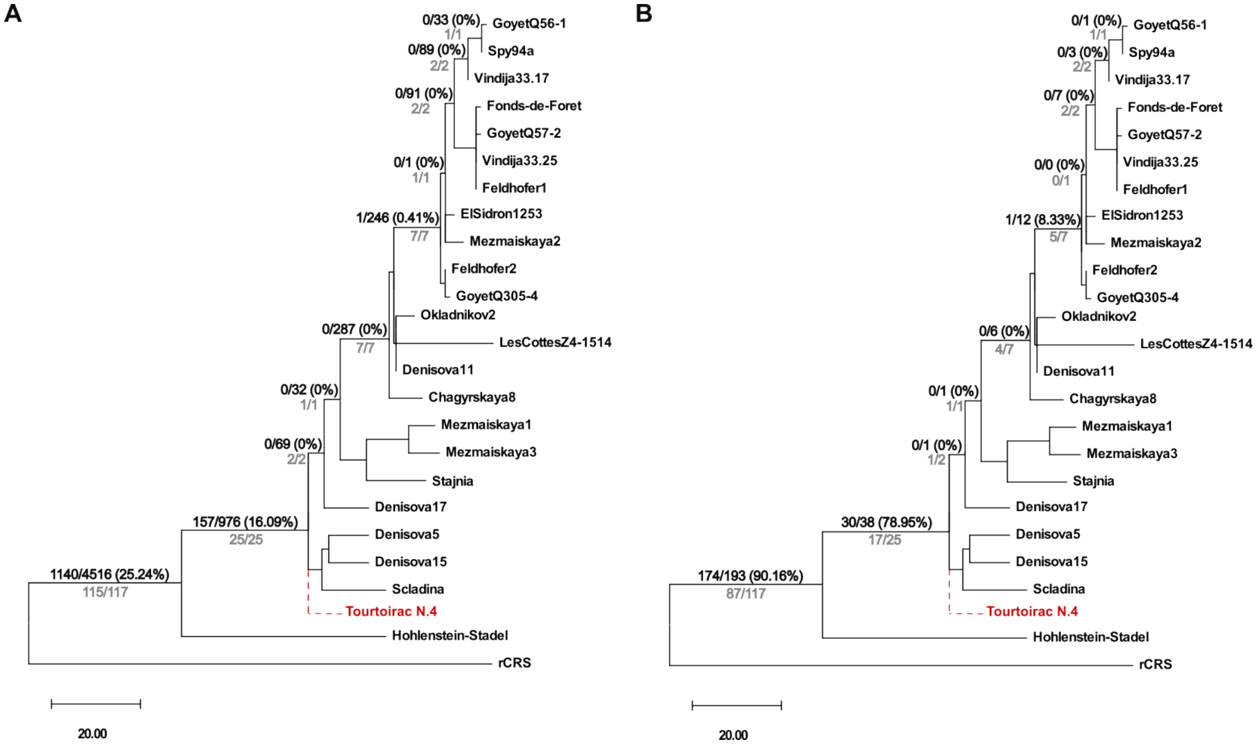

Fig. S15. Tourtoirac N.4 phylogenetic positioning according to the SNP analysis before (A) and after (B) PMD filtering. Values indicated above and below the nodes are as in Fig. S12.

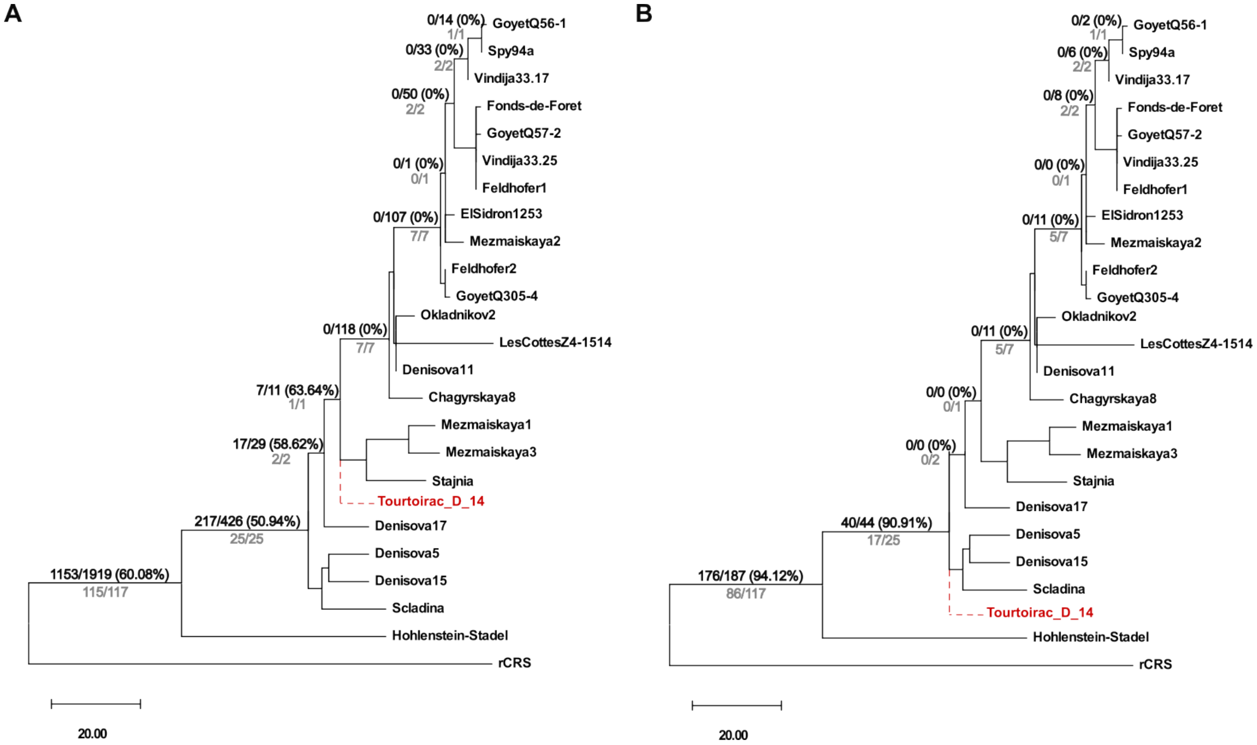

Fig. S16. Tourtoirac_D_14 phylogenetic positioning according to the SNP analysis before (A) and after (B) PMD filtering. Values indicated above and below the nodes are as in Fig. S12. For this individual the placement before and after PMD filtering changes. This is likely due to the low post-PMD coverage, which results in SNP-defining position for nodes Denisova 17 and Stajnia-Mezmaiskaya 1/3 to not be covered by any sequence. However, in the pre-PMD filtered data SNP positions defining both nodes are covered and show the presence of derived alleles alongside levels of modern human DNA contamination comparable to upstream nodes. Therefore, in Figure 2 we report the phylogenetic placement of Tourtoirac_D_14 with a dash line as shown here in panel A.

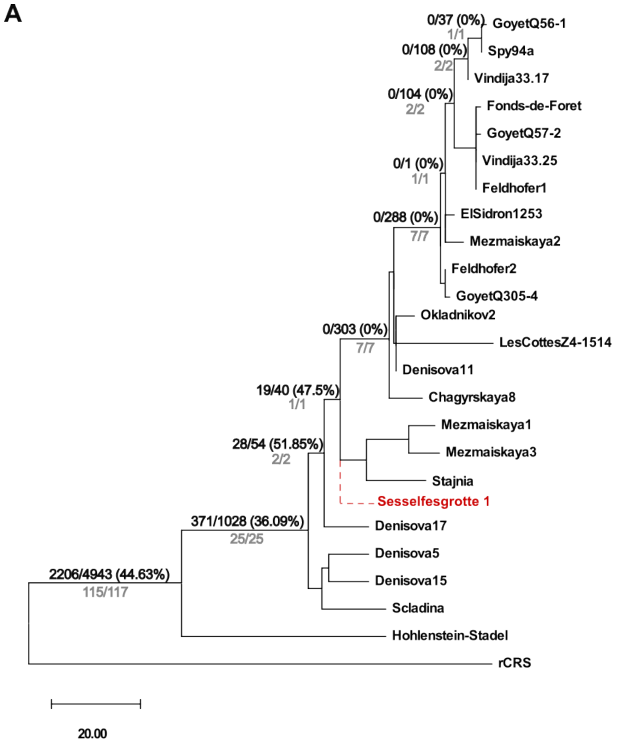

Fig. S17. Sesselfelsgrotte 1 phylogenetic positioning according to the SNP analysis before (A) and after (B) PMD filtering. Values indicated above and below the nodes are as in Fig. S12.

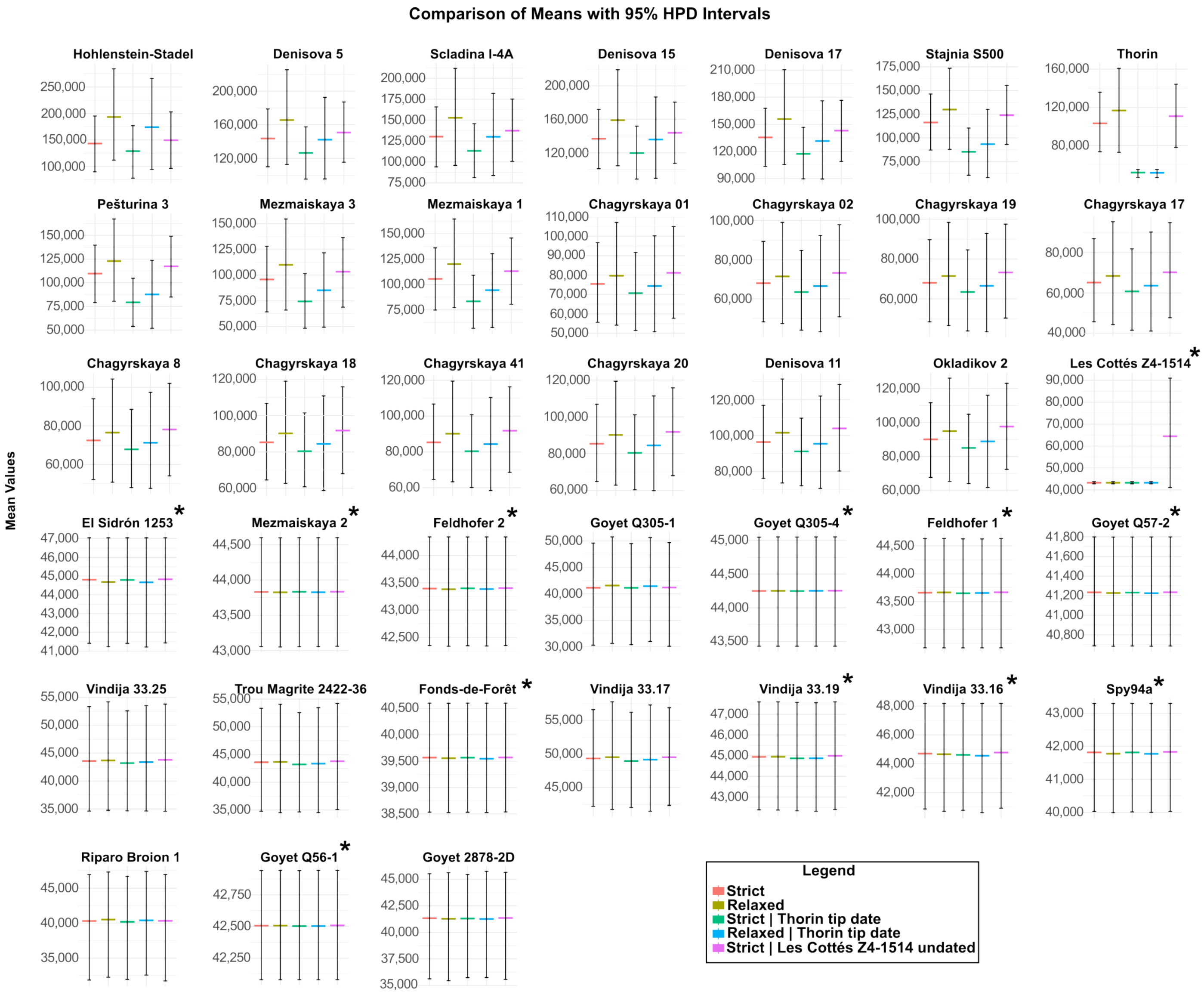

Fig. S18. Comparison of multiple BEAST runs. Boxplots show the mean molecular age estimates and their associated 95% Highest Posterior Density (HPD) intervals across different BEAST runs, each representing a distinct model parametrization. Individuals with an associated radiocarbon date are indicated with an asterisk next to their names. The first two runs correspond to the main Bayesian analyses described in the Materials and Methods, differing only in the choice of the molecular clock (strict vs. relaxed). The remaining runs incorporate modifications to either the tip dates or the priors. The third and fourth runs include Thorin as a tip date while the fifth run reports an analysis where Les Cottés Z4-1514 is treated as undated.

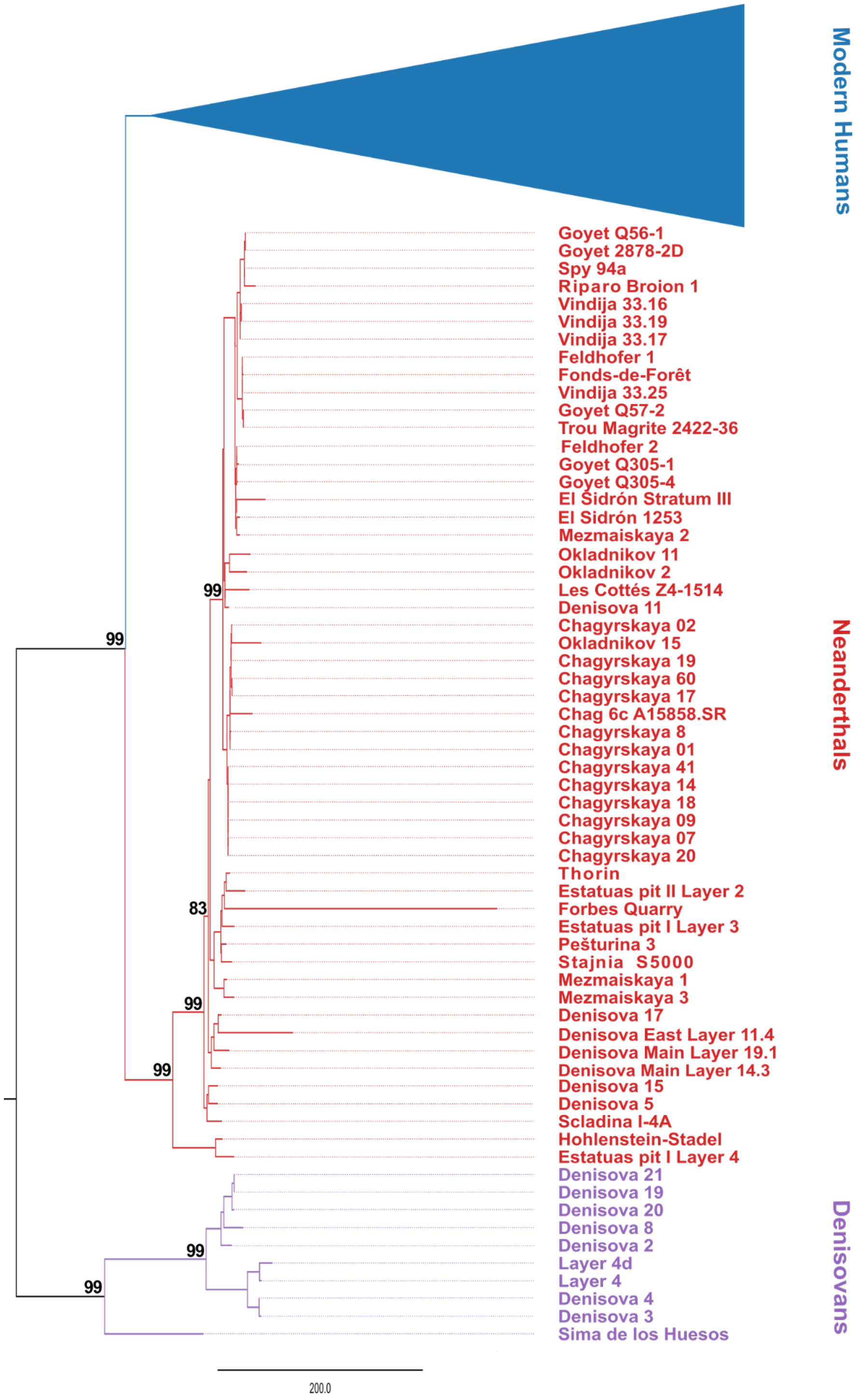

Fig. S19. Maximum Parsimony tree with the entire mtDNA of 49 published and four newly generated Neanderthals and 9 Denisovan mtDNA sequences with at least 85% covered positions (max 2600 Ns) plus 55 worldwide modern human mtDNAs, and Pan troglodytes used as outgroup. The tree was created with 90% partial deletion and 500 bootstrap iterations (bootstrap values reported only for the main nodes).

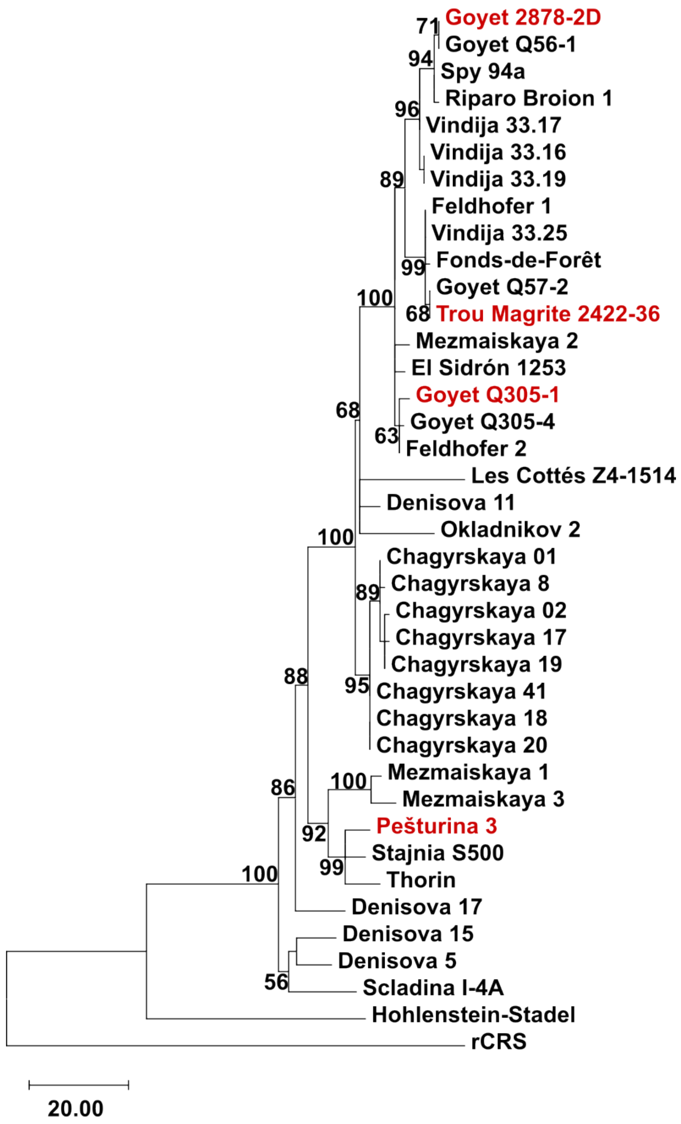

Fig. S20. Maximum Parsimony tree on the entire mtDNA of 34 published Neanderthal sequences with a maximum of 100 Ns, four newly generated Neanderthal sequences with at least 93% covered positions (indicated in red) and rCRS as outgroup. The tree was created with complete deletion and 500 bootstrap iterations (bootstrap values reported on the tree nodes).

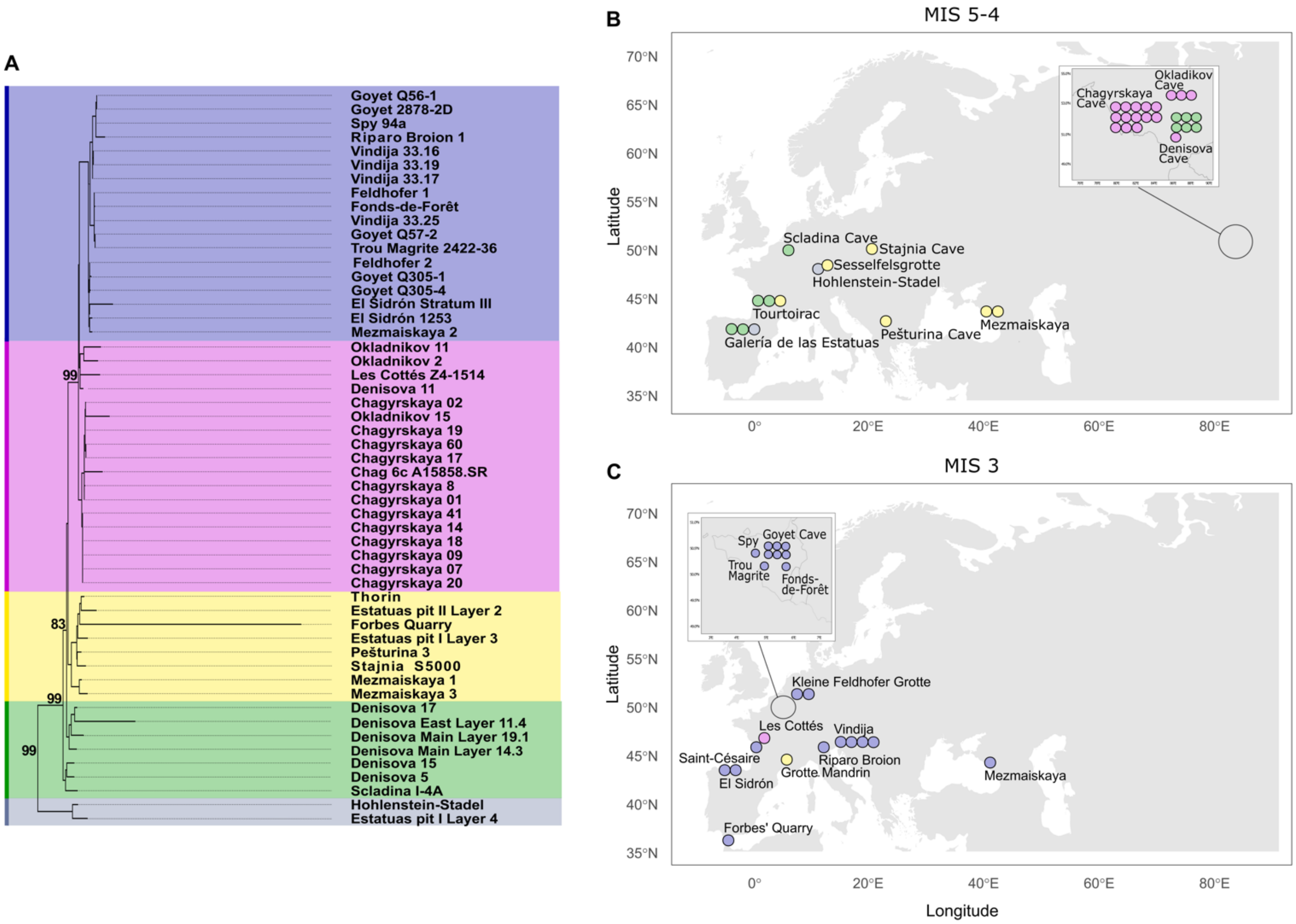

Fig. S21. Geographic distribution and phylogenetic relationships of Neanderthal mtDNA. (A) Maximum Parsimony tree including 49 previously published and 4 newly generated Neanderthal complete mtDNA sequences with at least 85% coverage and fewer than 2600 undetermined positions. Tree reconstruction was performed using 90% partial deletion and 500 bootstrap iterations. (B) Archaeological sites yielding Neanderthal mitochondrial DNA sequences dated to the Marine Isotope Stage 5-4 (MIS 5-4). Each circle represents an archaeological site, with the circle numbers proportional to the number of individuals analyzed. Colors indicate distinct mitochondrial lineages. (C) Same as panel A but restricted to sites dated to MIS 3. Circle numbers and colors correspond to the number of individuals and mtDNA lineages, respectively.

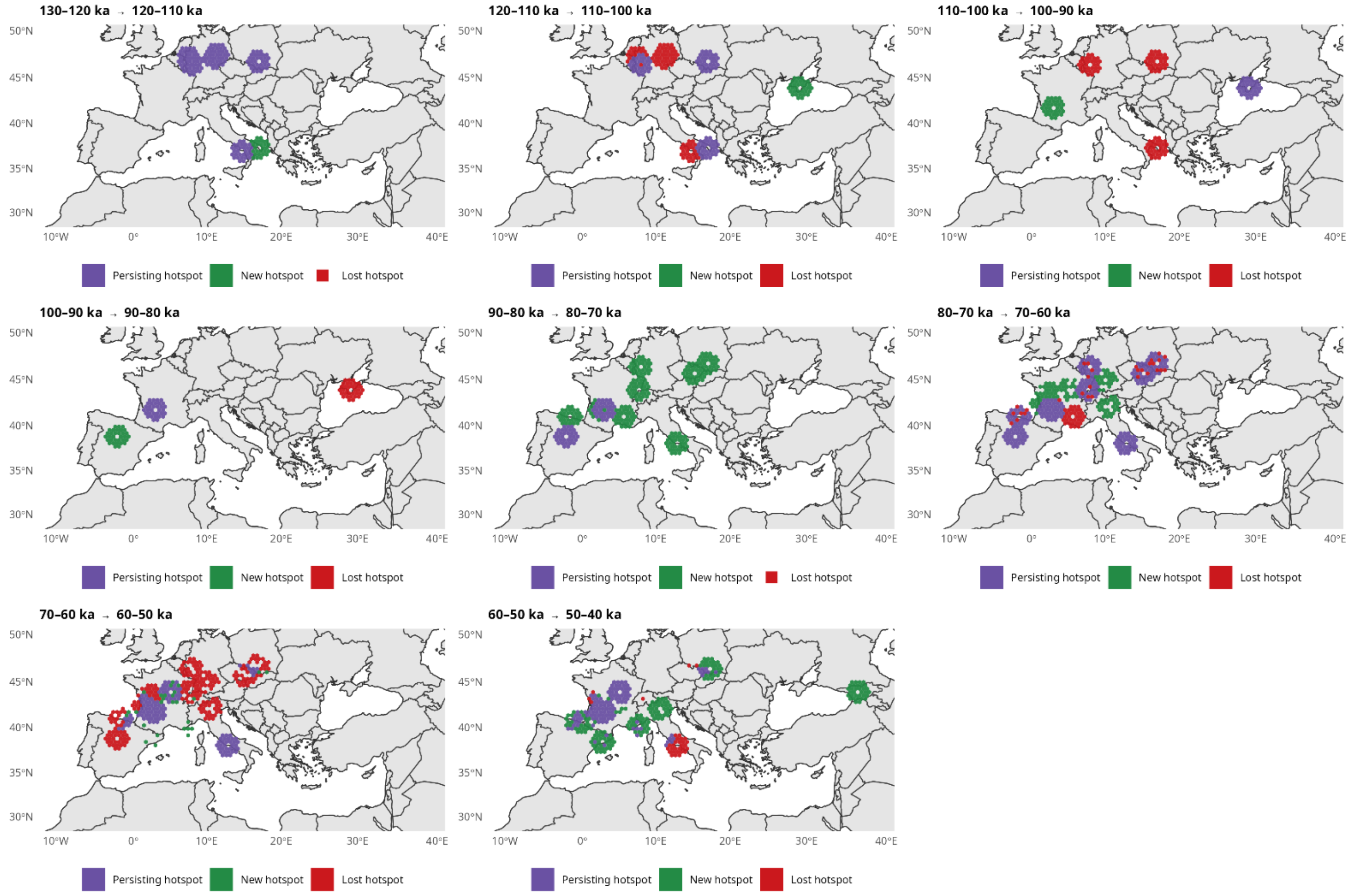

Fig. S22. Panel map visualizing slice-to-slice transitions. Each panel compares one time slice to the immediately preceding one, showing where hotspots persisted (violet), newly appeared (green), or were lost (red). Consistent color mapping across panels highlights temporal dynamics of clustering. From 80-70 ka onwards there are several hotspot losses while new and persisting hotspots are concentrated in western Europe, marking a consolidation of activity areas.

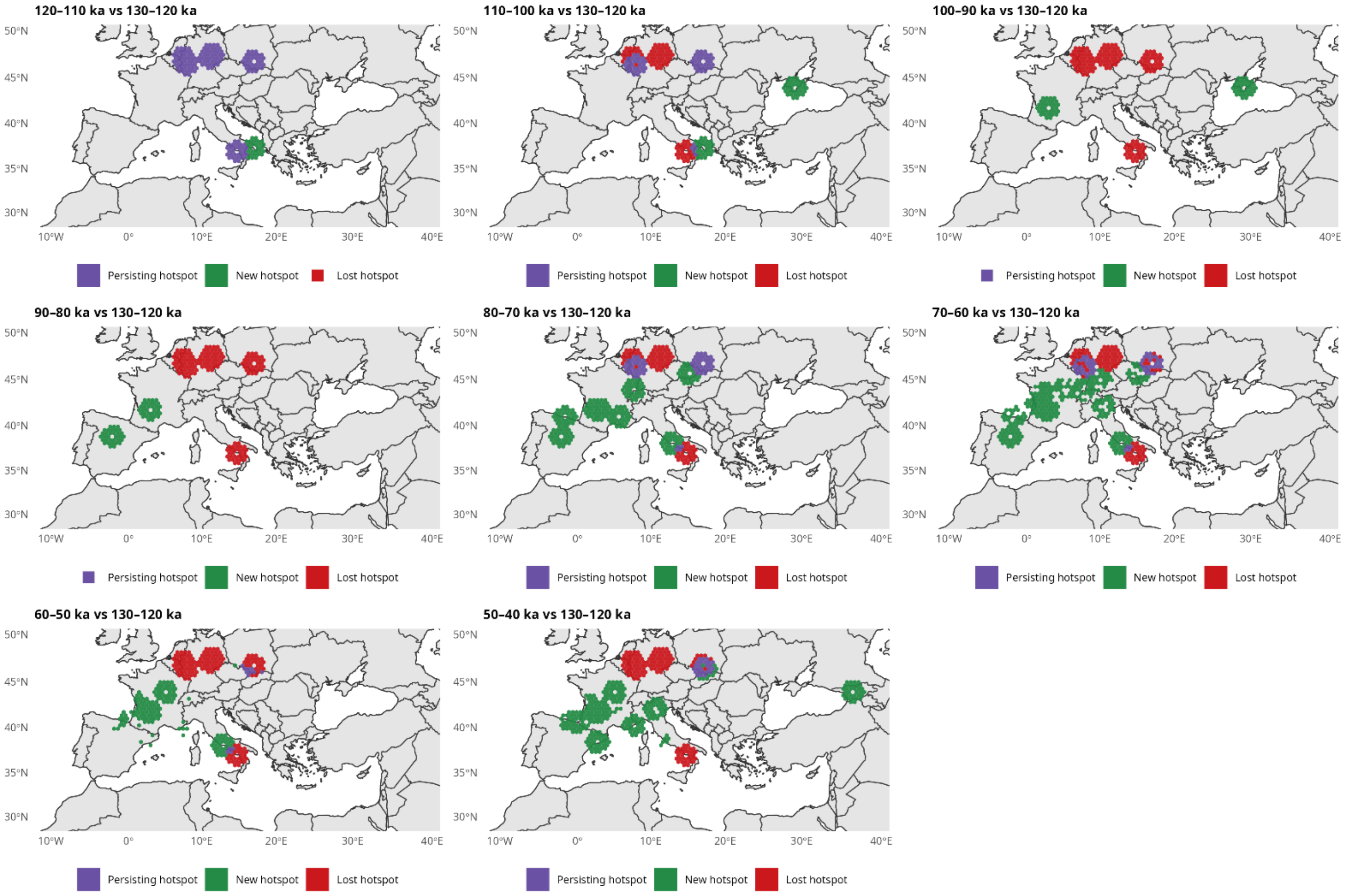

Fig. S23. Panel map visualizing slice transitions relative to a 130-120 ka baseline. Each map compares a given slice to the 130–120 ka baseline, using the same color scheme as Figure 23. Early slices largely maintain the baseline pattern, but from 80-70 ka onwards, several new hotspots emerge in western Europe, while hotspots in other regions tend to disappear.

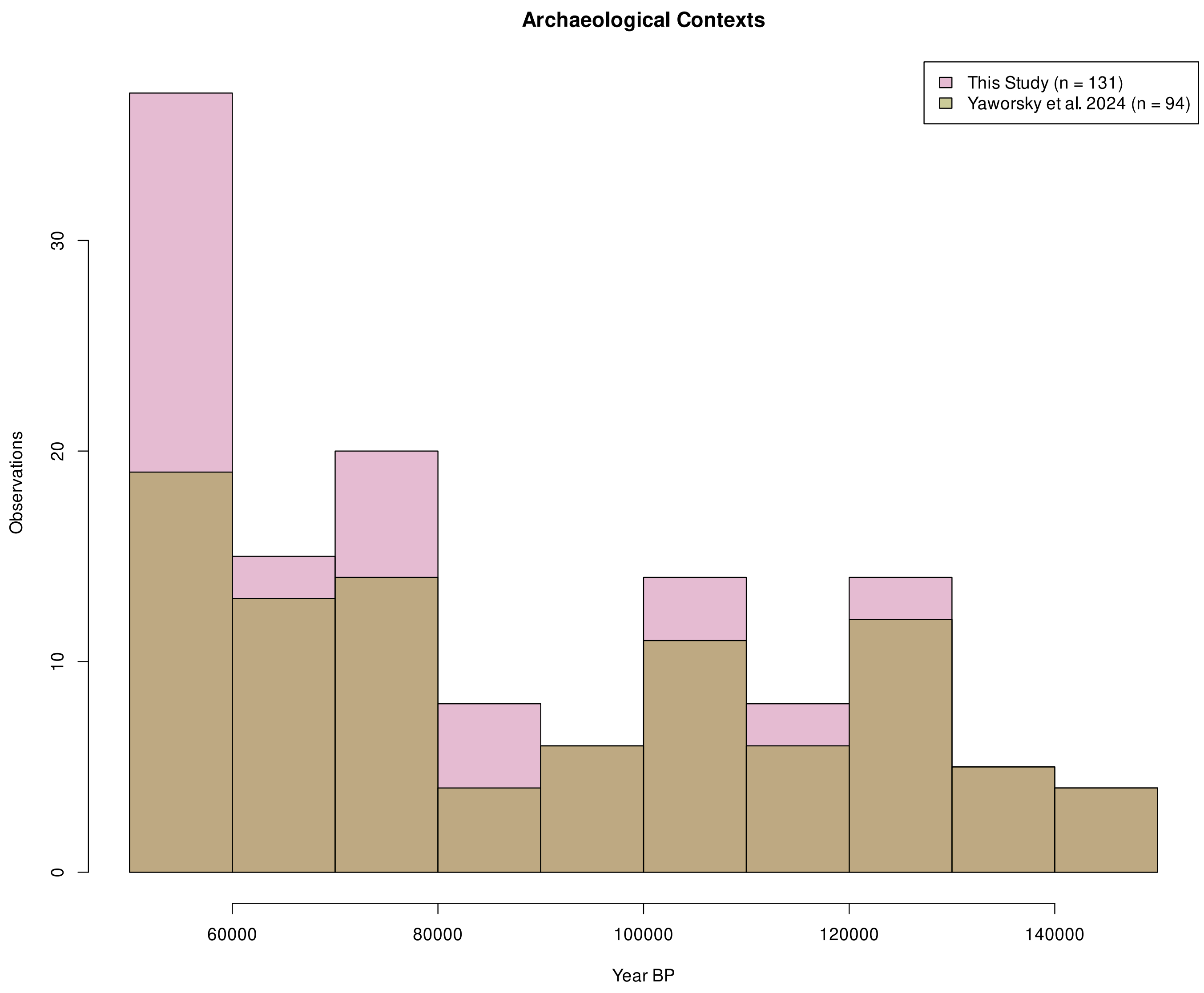

Fig. S24. Histograms showing the difference in dataset size between the original study by Yaworsky et al. (2024) and the present study.

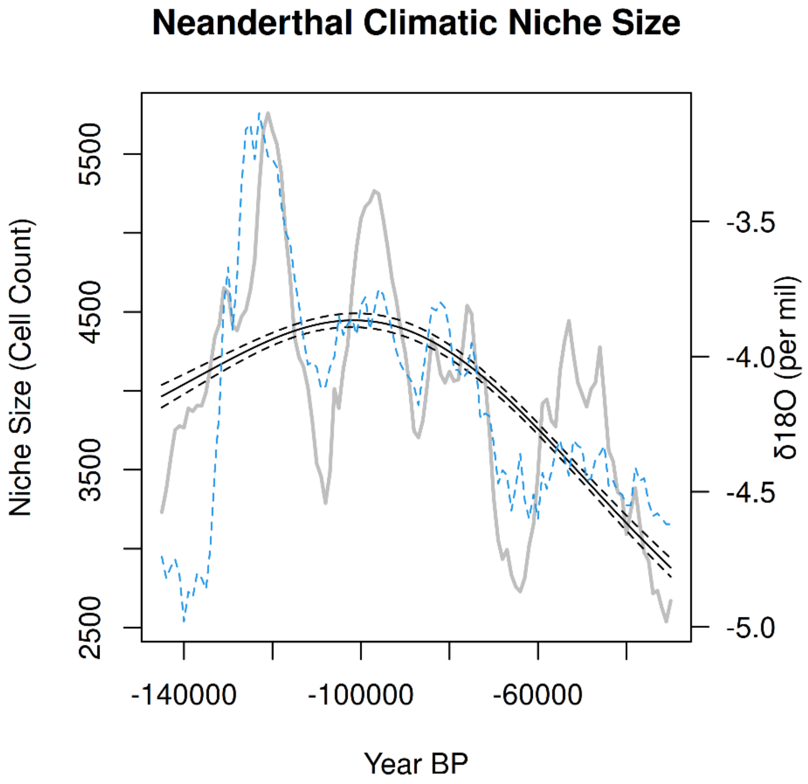

Fig. S25. Temporal changes in the size of the projected potential Neanderthal niche space over time (in years BP). The gray line shows millennial-scale fluctuations in niche size. The blue dashed line indicates variations in δ^{18}O as a proxy for paleoclimate. The black line represents the fitted Generalized Additive Mixed Model (GAMM) with associated 95% confidence intervals. Model statistics are included.

Table S1. Geographic, archaeological and skeletal information and summary statistics of newly reported mtDNAs of Neanderthal individuals before ancient DNA damage filtering. The mapped reads refer to the number of DNA sequences after the removal of duplicates, while damage 5'-end refers to the percentage of C-to-T substitutions at the 5' molecule terminus.

Country	Archaeological ID	Skeletal Element	Sampled amount (mg)	N. Sequenced Reads	N. Mapped Reads	Mean Coverage (X)	Damage 5'-end (%)	Average fragment length (bp)
Belgium	Goyet Q305-1	Right femur	77	40,356,947	32,998	146.0	29	73.3
Belgium	Goyet 2878-2D	Lower left P2	15	28,299,550	29,269	112.7	17	63.8
Belgium	Goyet D183-4	Left clavicle	33	30,888,723	12,436	41.4	19	55.2
Belgium	Trou Magrite 2422-36	Left femur	8.4	33,136,341	22,446	65.0	26	48.0
France	Tourtoirac N.1	Left tibia	50	71,232,316	23,212	98.6	12	70.4
France	Tourtoirac N.4	Femur	50	71,071,598	19,721	85.2	13	71.6
France	Tourtoirac_D_14	Distal hand phalanx	51	21,832,687	11,021	38.9	12	58.5
France	Saint-Césaire RPB_117	Left petrous	10	39,419,015	5,107	14.2	34	46.1
Germany	Sesselfelsgrotte 1	Right femur	10	73,807,769	24,430	94.7	10	43.6
Serbia	Pešturina 3	Upper right M1	36	41,037,815	26,125	76.6	58	48.6

Table S2. Contamination estimates measured with schmutzi (Renaud et al. 2015). Samples with schmutzi contamination estimates above 10% were PMD filtered.

Archaeological ID	Sample ID	ContDeam contamination estimates (%)	Schmutzi contamination estimates (%)	PMD filtered
Goyet Q305-1	GOY004.A	0 (0-5)	4 (3-5)	no
Goyet 2878-2D	GOY005.B	40 (39-41)	52 (51-53)	yes
Goyet D183-4	GOY008.A	24 (22 - 26)	36 (35-37)	yes
Trou Magrite 2422-36	TMA002.A	0 (0-0.5)	9 (8-10)	no
Saint-Césaire RPB_117	SCE001.A	12 (10-14)	26 (25-27)	yes
TTC N.1 (F7 layer 5)	TORT001.A	40 (39-41)	51 (50-52)	yes
TTC N. 4 (F7 layer 7)	TORT002.A	69 (68-70)	81 (80-82)	yes
TTC_D_14 (D5 A, US 23)	TORT004.A	26 (25-28)	43 (42-44)	yes
Sesselfelsgrotte 1	SESS001.A	45 (44-47)	61 (60-62)	yes
Pešturina 3	PES001.A	0 (0-0.5)	14 (13-15)	yes

Table S3. List of Neanderthals mtDNA included in each node for the SNP analysis.

Node	Neanderthals included
A	"Hohlenstein-Stadel", "Denisova5", "Denisova15", "Scladina I-4A", "Denisova17", "Chagyrskaya8", "ElSidron1253", "Feldhofer2", "GoyetQ305-4", "GoyetQ56-1", "Spy94a","Vindija33.17", "Mezmaiskaya2", "Feldhofer1", "Vindija33.25", "Fonds-de-Forêt", "GoyetQ57-2", "LesCottesZ4-1514", "Mezmaiskaya1", "Denisova11", "Stajnia S500", "Okladnikov2"
B	"Denisova5", "Denisova15", "Scladina I-4A", "Denisova17", "Chagyrskaya8", "ElSidron1253", "Feldhofer2", "GoyetQ305-4", "GoyetQ56-1", "Spy94a", "Vindija33.17", "Mezmaiskaya2", "Feldhofer1", "Vindija33.25", "Fonds-de-Forêt", "GoyetQ57-2", "LesCottesZ4-1514", "Mezmaiskaya1", "Denisova11", "Stajnia S500", "Okladnikov2"
C	"Denisova17", "Chagyrskaya8", "ElSidron1253", "Feldhofer2", "GoyetQ305-4", "Riparo", "GoyetQ56-1", "Spy94a", "Vindija33.16", "Vindija33.19", "Vindija33.17", "Mezmaiskaya2", "Feldhofer1", "Vindija33.25", "Fonds-de-Forêt", "GoyetQ57-2", "LesCottesZ4-1514", "Mezmaiskaya1", "Denisova11", "Stajnia S500", "Okladnikov2"
D	"Chagyrskaya8", "Chagyrskaya41", "ElSidron1253", "Feldhofer2", "GoyetQ305-4", "Riparo", "GoyetQ56-1", "Spy94a", "Vindija33.16", "Vindija33.19", "Vindija33.17", "Mezmaiskaya2", "Feldhofer1", "Vindija33.25", "Fonds-de-Forêt", "GoyetQ57-2", "LesCottesZ4-1514", "Mezmaiskaya1", "Denisova11", "Stajnia S500", "Okladnikov2"
E	"Chagyrskaya8", "ElSidron1253", "Feldhofer2", "GoyetQ305-4", "GoyetQ56-1", "Spy94a", "Vindija33.17", "Mezmaiskaya2", "Feldhofer1", "Vindija33.25", "Fonds-de-Forêt", "GoyetQ57-2", "LesCottesZ4-1514", "Denisova11", "Okladnikov2"
F	"ElSidron1253", "Feldhofer2", "GoyetQ305-4", "GoyetQ56-1", "Spy94a", "Vindija33.17", "Mezmaiskaya2", "Feldhofer1", "Vindija33.25", "Fonds-de-Forêt", "GoyetQ57-2"
G	"GoyetQ56-1", "Spy94a", "Vindija33.17", "Feldhofer1", "Vindija33.25", "Fonds-de-Forêt", "GoyetQ57-2", "ElSidron1253", "Mezmaiskaya2"
H	"GoyetQ56-1", "Spy94a","Vindija33.17", "Feldhofer1", "Vindija33.25", "Fonds-de-Forêt", "GoyetQ57-2"
I	"GoyetQ56-1", "Spy94a", "Vindija33.17"
J	"GoyetQ56-1", "Spy94a"

Table S4. Test for significant differences in the pairwise distances between Neanderthal groups (Wilcoxon test) with p-values both non-adjusted and adjusted for multiple testing with the Benjamini–Hochberg procedure.

Comparison	p-value	Adjusted p-value (BH)	Significant at p = 0.05
Late Neanderthals vs Goyet	0.5156	0.6382	No
Late Neanderthals vs Vindija	0.0218	0.0655	No
Late Neanderthals vs Feldhofer	0.6382	0.6382	No

Table S5. Number of nucleotide differences between nine complete mtDNA sequences deriving from Goyet in Belgium. The two newly reported sequences are indicated in red font.

	Goyet 2878-2D	GoyetQ 305-1	Goyet Q305-4	Goyet Q305-7	Goyet Q56-1	Goyet Q57-2	Goyet Q57-3	Goyet Q374a-1	Goyet Q57-1
**Goyet 2878-2D**									
**Goyet Q305-1**	14								
**Goyet Q305-4**	12	4							
**Goyet Q305-7**	0	15	13						
**Goyet Q56-1**	0	15	13	0					
**Goyet Q57-2**	14	13	11	14	14				
**Goyet Q57-3**	14	13	11	14	14	0			
**Goyet Q374a-1**	0	15	13	0	0	14	14		
**Goyet Q57-1**	14	10	8	11	11	0	0	11	

Table S6. Sample from prior analysis, which compares the prior and posterior distributions.

Parameter	Prior	Posterior
**Strict clock rate**	-1,727.35 (95% HPD: -1,745.05 to -1,705.57)	-30,947.21 (95% HPD: -31,002.58 to -30,894.56)
**Strick clock rate (Thorin tip-date)**	-1,727.74 (95% HPD: -1,777.59 to -1,668.26)	-30,947.98 (95% HPD: -31,004.64 to -30,890.09)
**Relaxed log normal clock rate**	-1,716.55 (95% HPD: -1739.36 to -1692.43)	-30,921.74 (95% HPD: -30,952 to -30,890)
**Relaxed log normal clock rate (Thorin tip-date)**	-1,717.17 (95% HPD: -1,740.85 to -1,692.86)	-30,922.51 (95% HPD: -30,954.04 to -30,831.02)
**Sample from prior**	-1,595.23 (95% HPD: -1,719.21 to -1,443.79)	-1,595.23 (95% HPD: -1,719.21 to -1,443.79)

Table S7. Radiocarbon dates of Neanderthal individuals used as time anchors in the BEAST analyses.

Specimen	Date	Lower	Upper	Uncalibrated	Author
**Feldhofer 1**	43,710	42,670	44,750	39,900±620 (ETH-20981)	(49)
**Feldhofer 2**	43,265	42,190	44,340	39,240±670 (ETH-19660)	(49)
**Vindija 33.16**	43,710	39,240	48,180	38,310±2,130 (U-n/a)	(50)
**El Sidrón 1253**	43,040	40,300	47,050	40,840±1,200 (Beta-192065)<br>37,300±830 (Beta-192066)<br>38,240±890 (Beta-192067)	(51)<br>(teeth and bone level III)
**Goyet Q56-1**	42,540	42,080	43,000	38,440+340-300(GrA-46170)	(5)
**Goyet Q57-2**	41,210	40,620	41,800	36,590+300-270(GrA-54024)	(5)
**Goyet Q305-4**	44,290	43,430	45,150	40,690+480,400(GrA-46176)	(5)
**Vindija 33.19**	45,300	42,000	47,600	older than 45.5 kBP with 95% probability (OxA-32278)	(52, 53)
**Mezmaiskaya 2**	43,780	42,960	44,600	39,700±1,100(OxA-21839)	(54)
**Les Cottés Z4-1514**	43230	42720	43740	(MAMS-26196)	(55)
**Spy 94a**	41,500	39,700	43,300	(OxA-X-2762-21)	(56)
**Fonds-de-Forêt 1**	39,500	38,400	40,600	(OxA-38322)	(56)

Table S8. Estimated molecular dates obtained with a strict and a log normal relaxed clock.

	Strict			Relaxed		
**Sequence Name**	**mean**	**lower 95% HPD interval**	**upper 95% HPD interval**	**mean**	**lower 95% HPD interval**	**upper 95% HPD interval**
**Denisova 5**	143,670	110,100	178,720	165,640	112,650	225,370
**Hohlenstein-Stadel**	143,130	89,766	195,080	193,250	111,760	284,210
**Denisova 15**	136,810	101,200	171,780	158,950	104,510	219,160
**Denisova 17**	135,470	103,350	167,700	155,760	105,210	210,460
**Scladina I-4A**	130,180	94,032	165,750	152,680	95,780	211,930
**Stajnia S5000**	116,250	87,163	146,260	129,920	87,828	173,440
**Pešturina 3**	109,690	78,981	139,680	122,930	80,501	167,390
**Mezmaiskaya 1**	105,460	74,733	136,100	120,120	77,104	164,560
**Thorin**	103,230	73,522	135,740	116,670	72,924	160,770
**Denisova 11**	96,278	76,051	116,790	101,500	73,477	131,460
**Mezmaiskaya 3**	95,750	64,220	127,870	109,930	65,955	154,500
**Okladnikov 2**	90,085	67,591	111,770	94,970	65,288	126,310
**Chagyrskaya 41**	85,351	64,595	106,770	90,167	63,320	119,500
**Chagyrskaya 18**	85,325	64,581	106,810	90,220	62,696	119,020
**Chagyrskaya 20**	85,315	64,734	106,820	90,182	62,807	119,290
**Chagyrskaya 01**	75,478	55,681	96,737	79,700	54,172	107,190
**Chagyrskaya 8**	72,498	52,256	94,096	76,542	50,790	104,350
**Chagyrskaya 19**	68,047	48,549	89,694	71,462	46,605	98,339
**Chagyrskaya 02**	68,020	48,251	89,299	71,492	47,403	99,088
**Chagyrskaya 17**	65,189	45,661	87,048	68,453	44,251	95,459
**Vindija 33.17**	49,315	42,172	56,603	49,508	41,712	57,762
**Vindija 33.19**	44,941	42,382	47,597	44,948	42,375	47,598
**El Sidrón 1253**	44,808	41,412	47,050	44,684	41,232	47,050
**Vindija 33.16**	44,700	40,857	48,180	44,655	40,694	48,178
**Goyet Q305-4**	44,249	43,431	45,047	44,252	43,430	45,051
**Mezmaiskaya 2**	43,831	43,057	44,600	43,826	43,051	44,598
**Feldhofer 1**	43,662	42,671	44,632	43,665	42,670	44,633
**Trou Magrite 2422-36**	43,600	34,754	53,341	43,656	34,492	54,071
**Vindija 33.25**	43,593	34,617	53,328	43,690	34,740	54,180
**Feldhofer 2**	43,394	42,351	44,340	43,382	42,337	44,340
**Les Cottés Z4-1514**	43,249	42,776	43,740	43,250	42,777	43,740
**Goyet Q56-1**	42,506	42,080	42,941	42,506	42,080	42,942
**Spy 94a**	41,816	40,026	43,300	41,777	39,989	43,300
**Goyet 2878-2D**	41,323	35,623	45,510	41,271	35,453	45,605
**Goyet Q57-2**	41,234	40,687	41,800	41,227	40,683	41,799
**Goyet Q305-1**	41,156	30,308	49,577	41,570	30,646	50,745
**Riparo Broion**	40,305	31,869	46,927	40,511	32,271	47,310
**Fonds-de-Forêt 1**	39,568	38,534	40,600	39,557	38,527	40,598

Table S9. Divergence dates obtained via molecular dating in BEAST.

Most recent common ancestor	Mean	Median	95% HPD interval	
**Modern Humans and Neanderthals**	397,150	396,580	349,150	444,980
**Modern Humans**	136,380	135,770	112,540	161,040
**Neanderthals**	274,770	274,080	235,120	315,880
**Late Neanderthals**	65,483	65,048	56,417	75,684

Table S10. Summary of statistically significant for local clusters of Neanderthal site density for each 10,000-year time slice between 130–40 ka. Each row lists the time interval (Slice), the total number of hexagons in the analysis window (Cells (n)), the number classified as hotspots (FDR ≤ 0.10; Hotspot Total), and the total number of sites assigned to those hexes (Site Total). The hotspot area proxy (Hotspot Area Total (km2)) is derived from hotspot count × hex area, providing a measure of the areal footprint of clustering through time. The results show a contraction in hotspot area from 130–110 ka to 90-80 ka followed by renewed clustering after ~80 ka.

Slice	Cells (n)	Hotspot Total	Site Total	Hotspot Area Total (km2)
130,000 – 120,000	8839	152	8847	329089.65
120,000 – 110,000	8839	179	8848	387546.36
110,000 – 100,000	8839	129	8844	279293.19
100,000 – 90,000	8839	66	8841	142894.19
90,000 – 80,000	8839	66	8841	142894.19
80,000 – 70,000	8839	293	8850	634363.60
70,000 – 60,000	8839	334	8857	723131.21
60,000 – 50,000	8839	150	8861	324759.52
50,000 – 40,000	8839	296	8872	640858.79

Table S11. Structured permutation tests evaluating whether the observed rise in hotspots in France exceeds random expectation. For each time slice (Slice), the table lists the observed number of new French hotspots (Obs, z ≥ 1.96 relative to baseline 130–120 ka), the mean value expected under random permutation of temporal labels (Mean Null), and the resulting one-sided p-value (P-value (One Sided)). Significant values (p < 0.05) for 60–50 ka and 50–40 ka demonstrate that the late concentration of hotspots in France cannot be explained by spatial sampling bias alone.

Slice	Obs	Mean Null	P-value (One Sided)
120,000 – 110,000	0	53.04	1.00
110,000 – 100,000	0	10.47	1.00
100,000 – 90,000	33	11.77	0.21
90,000 – 80,000	33	17.46	0.30
80,000 – 70,000	94	56.99	0.11
70,000 – 60,000	127	85.11	0.07
60,000 – 50,000	154	90.04	0.01
50,000 – 40,000	117	70.92	0.04

Table S12. Permutation results testing whether each time slice contains more hotspots than expected by chance. For each slice (Slice), the observed number of Gi* hotspots (Hotspot Obs, z ≥ 1.96) is compared to the permutation mean and standard deviation (Mean, SD) and to the 5–95 % quantile range (Q05, Q95). The one-sided probability (P-value (One Sided)) expresses the likelihood that the observed value could arise under random redistribution of site counts.

Slice	Hotspot Obs	Mean	SD	Q05	Q95	P-value (One Sided)
130,000 – 120,000	152	190.59	9.05	173	201.00	0.99
120,000 – 110,000	179	222.12	10.31	202	234.00	0.99
110,000 – 100,000	129	127.15	7.66	112	135.00	0.59
100,000 – 90,000	66	63.92	4.97	52	68.00	0.49
90,000 – 80,000	66	63.90	5.07	52	68.05	0.50
80,000 – 70,000	293	315.04	13.00	291	332.00	0.94
70,000 – 60,000	392	438.53	16.67	409	462.00	0.99
60,000 – 50,000	416	468.88	17.65	437	494.00	0.99
50,000 – 40,000	300	295.96	15.16	272	321.00	0.41

Table S13. Rarefaction-based comparison of longitudinal spread relative to the 80–70 ka baseline. All younger time slices were subsampled to match the number of localities observed in the 80-70 ka slice (Rarefied n = 60), and the procedure was repeated 1000 times. Full longitude range denotes the maximum west-east extent of sites (min-max longitude), while the central 95% range represents the longitude span containing the central 95% of sites and provides a robust measure of the core geographic footprint. Reported range values are medians across rarefaction replicates. p(full > 80-70) and p(95% > 80-70) are one-sided Monte Carlo p-values testing whether the rarefied longitudinal ranges exceed those observed in the 80-70 ka baseline. New longitude bins indicate the median number of previously unoccupied 2° longitude bins that become occupied relative to the baseline under rarefaction.

Time slice (ka)	Rarefied n	Full longitude range (median, °)	p(full > 80–70)	Central 95% range (median, °)	p(95% > 80–70)	New longitude bins (median)
80–70	60	47.2	–	32.3	–	0
70–60	60	47.2	0.502	40.0	0.009	2
60–50	60	51.9	0.045	43.5	0.011	3
50–40	60	51.9	0.022	47.0	0.004	3

Dataset S1 (separate file). Laboratory procedures.

Dataset S2 (separate file): A) Neanderthal and Denisovan mtDNA Dataset; B) TempEst Maximum Parsimony Results; C) Neanderthal mtDNA SNP Positions.

SI References

1. M. Toussaint, S. Pirson, Neandertal studies in Belgium: 2000–2005. *Period. Biol.* **108**, 373–387 (2006).
2. M. Toussaint, 1997-2005 research in the caves of Goyet (Gesves, Province of Namur, Belgium). Tongeren Neandertal symposium excursion, 19 September 2004 in Proceedings of the International Conference held in the Gallo-Roman Museum in Tongeren, 17-19 September 2004, B. Demarsin, M. Otte, Dir. (ERAUL 117, Liège, ATVATVCA 2, Tongeren, 2006) pp. 115–134.
3. M. Germonpré, A reconstruction of the spatial distribution of the faunal remains from Goyet, Belgium. *Notae praehistoricae* **21**, 57–65 (2001).
4. H. Rougier *et al.*, The Troisième caverne of Goyet (Belgium): An exceptional site with both Neandertal and Upper Paleolithic human remains. *PESHE* **5**, 211 (2016).
5. H. Rougier *et al.*, Neandertal cannibalism and Neandertal bones used as tools in Northern Europe. *Sci. Rep.* **6**, 29005 (2016).
6. P. J. Reimer *et al.*, The IntCal20 Northern Hemisphere radiocarbon age calibration curve (0-55 cal kBP). *Radiocarbon* **62**, 725–757 (2020).
7. M. T. Krajcarz, M. Krajcarz, D. G. Drucker, H. Bocherens, Bone/tooth sampling and cleaning for collagen extraction. protocols.io [Preprint] (2024). https://dx.doi.org/10.17504/protocols.io.eq2lyw6mrvx9/v1 (accessed 24 July 2025).
8. M. T. Krajcarz, D. G. Drucker, M. Krajcarz, H. Bocherens, Collagen extraction from pretreated bone/tooth samples. protocols.io [Preprint] (2024). https://dx.doi.org/10.17504/protocols.io.8epv5rrp4g1b/v1 (accessed 24 July 2025).
9. S. H. Ambrose, Preparation and characterization of bone and tooth collagen for isotopic analysis. *J. Archaeol. Sci.* **17**, 431–451 (1990).
10. M. J. DeNiro, Postmortem preservation and alteration of in vivo bone collagen isotope ratios in relation to palaeodietary reconstruction. *Nature* **317**, 806–809 (1985).
11. É. Dupont, Découverte d’objets gravés et sculptés dans le Trou Magrite à Pont-à-Lesse. *Bull. Acad. roy. Sci. Belg.* **24**, 129–132 (1867).
12. M. Otte, L. G. Straus, Le Trou Magrite : fouilles 1991-1992. Résurrection d’un site classique en Wallonie (ERAUL 69, Liège, 1995).
13. E.-L. Jimenez, A. Smolderen, I. Jadin, M. Germonpré, Exhumation de la collection faunique d’Édouard Dupont provenant du Trou Magrite (Pont-à-Lesse) Quelles données et quelles perspectives pour une collection du XIXe siècle? *Notae Praehistoricae* **36**, 167–190 (2016).
14. F. Twiesselmann, “Belgium” in Catalogue of Fossil Hominids - Part 2: Europe, K. P. Oakley, B. G. Campbell, T. I. Molleson, Eds. (Trustees of the British Museum (Natural History), 1971), pp. 5–13.
15. F. Lévêque, A. M. Backer, M. Guilbaud, Context of a late Neandertal. Implications of multidisciplinary research for the transition to Upper Paleolithic adaptations at Saint-Césaire, Charente-Maritime, France (Monographs in World Archaeology 16, Prehistory Press, 1993).
16. P. Colombet, P. Bayle, I. Crevecoeur, J. G. Ferrié, B. Maureille, New Mousterian neonates from the South-West of France (Saint-Césaire, Charente-Maritime). *PESHE* **1**, 57 (2012).
17. D. Todisco *et al.*, A multiscalar and multiproxy geoarchaeological approach to site formation processes at the Middle and Upper Palaeolithic site of La Roche-à-Pierrot, Saint-Césaire, France. *Quat. Sci. Rev.* **315**, 108218 (2023).
18. J. Parrot, La grotte de Tourtoirac (Dordogne). *Bull. Mém. Soc. Anthropol. Paris* **8**, 140–141 (1873).
19. D. Raoul, Note sur les niveaux aurignaciens et moustériens de la grotte de Tourtoirac (Dordogne). *Bull. Soc. préhist. fr.* **29**, 527–528 (1932).
20. F. Bordes, Aquitaine. *Gallia préhistoire* **13**, 485–511 (1970).
21. F. Bordes, Aquitaine. *Gallia préhistoire* **15**, 487–497 (1972).
22. F. Bordes, Aquitaine. *Gallia préhistoire* **17**, 617–628 (1974).
23. L. Doyon *et al.*, Rapport d’activités - Opération 2022 (triennale 2022-2024) - Abri Tourtoirac, lieux-dit : Grotte de Tourtoirac / Bel-Air (DRAC Nouvelle-Aquitaine, SRA, 2022).

24. I. Crevecoeur *et al.,* Découverte de restes humains néandertaliens à l'abri Tourtoirac (Dordogne). *Bull. Mém. Soc. Anthropol. Paris* **36S**, S21 (2024).
25. D. Mihailović *et al.,* Neanderthal settlement of the Central Balkans during MIS 5: Evidence from Pešturina Cave, Serbia. *Quat. Int.* **610**, 1–19 (2022).
26. J. A. Lindal, P. Radović, D. Mihailović, M. Roksandic, Postcranial hominin remains from the Late Pleistocene of Pešturina Cave (Serbia). *Quat. Int.* **542**, 9–14 (2020).
27. P. Radović, J. Lindal, D. Mihailović, M. Roksandic, The first Neanderthal specimen from Serbia: Maxillary first molar from the Late Pleistocene of Pešturina Cave. *J. Hum. Evol.* **131**, 139–151 (2019).
28. G. Freund, Sesselfelsgrotte I: Grabungsverlauf und Stratigraphie (Saarbrücker Druckerei und Verlag, 1998).
29. T. Rathgeber, Fossile Menschenreste aus der Sesselfelsgrotte im unteren Altmühltal (Bayern, Bundesrepublik Deutschland). *Quartär* **53/54**, 33–59 (2006).
30. D. Richter *et al*., "Luminescence Dating of the Middle/Upper Palaeolithic Sites 'Sesselfelsgrotte' and 'Abri I am Schulerloch', Altmühltal, Bavaria" in Neanderthals and Modern Humans – Discussing the Transition. Central and Eastern Europe from 50.000 – 30.000 B.P., J. Orschiedt, G.-Ch. Weniger, Eds. (Universität zu Köln, 2000), pp. 30–41.
31. J. Richter, Sesselfelsgrotte III - Der G-Schichten Komplex der Sesselfelsgrotte. Zum Verständnis des Micoquien (Quartär Bibliothek 7, Saarbrücker Druckerei und Verlag, 1997).
32. J. Richter, Die 14C-Daten aus der Sesselfelsgrotte und die Zeitstellung des Micoquien/MMO. *Germania* **80**, 1–22 (2002).
33. P. Korlević *et al*., Reducing microbial and human contamination in DNA extractions from ancient bones and teeth. *Biotechniques* **59**, 87–93 (2015).
34. J. Dabney *et al.,* Complete mitochondrial genome sequence of a Middle Pleistocene cave bear reconstructed from ultrashort DNA fragments. *Proc. Natl. Acad. Sci. U.S.A.* **110**, 15758–15763 (2013).
35. M.-T. Gansauge, A. Aximu-Petri, S. Nagel, M. Meyer, Manual and automated preparation of single-stranded DNA libraries for the sequencing of DNA from ancient biological remains and other sources of highly degraded DNA. *Nat. Protoc.* **15**, 2279–2300 (2020).
36. M.-T. Gansauge *et al*., Single-stranded DNA library preparation from highly degraded DNA using T4 DNA ligase. *Nucleic Acids Res.* **45**, e79 (2017).
37. P. Skoglund *et al*., Separating endogenous ancient DNA from modern day contamination in a Siberian Neandertal. *Proc. Natl. Acad. Sci. U.S.A.* **111**, 2229–2234 (2014).
38. Q. Fu *et al*., A Revised Timescale for Human Evolution Based on Ancient Mitochondrial Genomes. *Curr. Biol.* **23**, 553–559 (2013).
39. A. Furtwängler *et al*., Ratio of mitochondrial to nuclear DNA affects contamination estimates in ancient DNA analysis. *Sci. Rep.* **8**, 14075 (2018).
40. A. Peltzer *et al*., EAGER: efficient ancient genome reconstruction. *Genome Biol.* **17**, 60 (2016).
41. H. Jónsson, A. Ginolhac, M. Schubert, P. L. F. Johnson, L. Orlando, mapDamage2.0: fast approximate Bayesian estimates of ancient DNA damage parameters. *Bioinformatics* **29**, 1682–1684 (2013).
42. G. Renaud, V. Slon, A. T. Duggan, J. Kelso, Schmutzi: estimation of contamination and endogenous mitochondrial consensus calling for ancient DNA. *Genome Biol.* **16**, 224 (2015).
43. P. Skoglund *et al*., Separating endogenous ancient DNA from modern day contamination in a Siberian Neandertal. *Proc. Natl. Acad. Sci. U.S.A.* **111**, 2229–2234 (2014).
44. M. Meyer *et al*., Nuclear DNA sequences from the Middle Pleistocene Sima de los Huesos hominins. *Nature* **531**, 504–507 (2016).
45. S. Kumar, G. Stecher, M. Li, C. Knyaz, K. Tamura, MEGA X: Molecular Evolutionary Genetics Analysis across Computing Platforms. *Mol. Biol. Evol.* **35**, 1547–1549 (2018).
46. A. Bossoms Mesa *et al*., Resolving the relatedness of the Neandertals from the Troisième caverne of Goyet using ancient DNA. *PaleoAnthropology* **2022**, 437 (2022).
47. J. Heled, A. J. Drummond, Bayesian inference of population size history from multiple loci. *BMC Evol. Biol.* **8**, 289 (2008).

48. A. Rambaut, T. T. Lam, L. M. Carvalho, O. G. Pybus, Exploring the temporal structure of heterochronous sequences using TempEst (formerly Path-O-Gen). *Virus Evol.* **2**, vew007 (2016).
49. R. W. Schmitz *et al.*, The Neandertal type site revisited: Interdisciplinary investigations of skeletal remains from the Neander Valley, Germany. *Pro. Natl. Acad. Sci. U.S.A.* **99**, 13342–13347 (2002).
50. D. Serre *et al.*, No Evidence of Neandertal mtDNA Contribution to Early Modern Humans. *PLoS Biol.* **2**, e57 (2004).
51. C. Lalueza-Fox *et al.*, Neandertal Evolutionary Genetics: Mitochondrial DNA Data from the Iberian Peninsula. *Mol. Biol. Evol.* **22**, 1077–1081 (2005).
52. T. Devièse *et al.*, Direct dating of Neanderthal remains from the site of Vindija Cave and implications for the Middle to Upper Paleolithic transition. *Proc. Natl. Acad. Sci. U.S.A.* **114**, 10606–10611 (2017).
53. K. Prüfer *et al.*, A high-coverage Neandertal genome from Vindija Cave in Croatia. *Science* **358**, 655–658 (2017).
54. R. Pinhasi, T. F. G. Higham, L. V. Golovanova, V. B. Doronichev, Revised age of late Neanderthal occupation and the end of the Middle Paleolithic in the northern Caucasus. *Proc. Natl. Acad. Sci. U.S.A.* **108**, 8611–8616 (2011).
55. M. Hajdinjak *et al.*, Reconstructing the genetic history of late Neanderthals. *Nature* **555**, 652–656 (2018).
56. T. Devièse *et al.*, Reevaluating the timing of Neanderthal disappearance in Northwest Europe. *Proc. Natl. Acad. Sci. U.S.A.* **118**, e2022466118 (2021).
57. P. M. Yaworsky, E. S. Nielsen, T. K. Nielsen, The Neanderthal niche space of Western Eurasia 145 ka to 30 ka ago. *Sci. Rep.* **14**, 7788 (2024).
